# Effectiveness of a School‐Centered Intervention for Myopia and Obesity Comorbidity in Children: A Cluster‐Randomized Trial

**DOI:** 10.1002/mco2.71092

**Published:** 2026-10-25

**Authors:** Jing Li, Di Shi, Yaqi Wang, Yihang Zhang, Jiajia Dang, Yunfei Liu, Jieyu Liu, Ziyue Chen, Xinxin Wang, Jieyun Song, Jun Ma, Randall S. Stafford, Yanhui Dong, Yi Song

**Affiliations:** ^1^ Institute of Child and Adolescent Health School of Public Health Peking University Beijing China; ^2^ National Health Commission Key Laboratory of Reproductive Health Peking University Beijing China; ^3^ Linyi University Linyi Shandong China; ^4^ Stanford Prevention Research Center Palo Alto California USA

**Keywords:** cluster‐randomized trial, comorbidity, myopia, obesity, RE‐AIM, school‐based intervention

## Abstract

The rising comorbidity of myopia and overweight/obesity (OWOB) in children shares modifiable behavioral pathways, yet evidence for integrated prevention remains limited. We conducted a cluster‐randomized, hybrid type I effectiveness‐implementation trial in 36 pair‐matched Beijing schools. Of 4991 enrolled students in Grades 1–4 and 7, 3426 (68.6%) completed the 12‐month follow‐up. The school‐centered “22510SS” intervention targeted outdoor activity, screen time, diet, sleep, and health monitoring. No statistically significant between‐group differences were observed in spherical equivalent or BMI change, or in the prevalence of myopia, OWOB, or their comorbidity. The intervention improved the overall behavior‐improvement rate (39.7% vs. 33.7%; OR, 1.34; 95% CI, 1.00–1.80). In a prespecified analysis, students in Grades 1–4 showed slower myopia progression (OR, 0.46; 95% CI, 0.25–0.86); an exploratory analysis suggested benefits among children from less supportive family environments. Reach, Effectiveness, Adoption, Implementation, and Maintenance (RE‐AIM) evaluation showed 100% fidelity for school‐based activities, approximately 60% completion of family‐facing health‐tip dissemination, an incremental cost of ¥215.43 per participant, and a Program Sustainability Assessment Tool (PSAT) score of 6.10/7. The intervention was feasible and improved targeted behaviors but did not produce significant population‐level clinical effects within 12 months. Stronger family engagement and longer follow‐up may be needed.

Trial Registration: NCT05275959

## Graphical abstract

This cluster randomized controlled trial assessed the effectiveness and implementation of a digital‐assisted school‐family intervention (“22510SS”) targeting myopia and obesity among 4991 students in Beijing. Over 12 months, the multicomponent program significantly improved key health behaviors, especially among younger children and those from less supportive family environments, although no significant differences were observed in refractive outcomes or BMI. Implementation evaluation via the RE‐AIM framework highlighted the program's low per‐capita cost and high school‐based fidelity, despite challenges in family engagement, suggesting a feasible model for school‐centered health promotion.

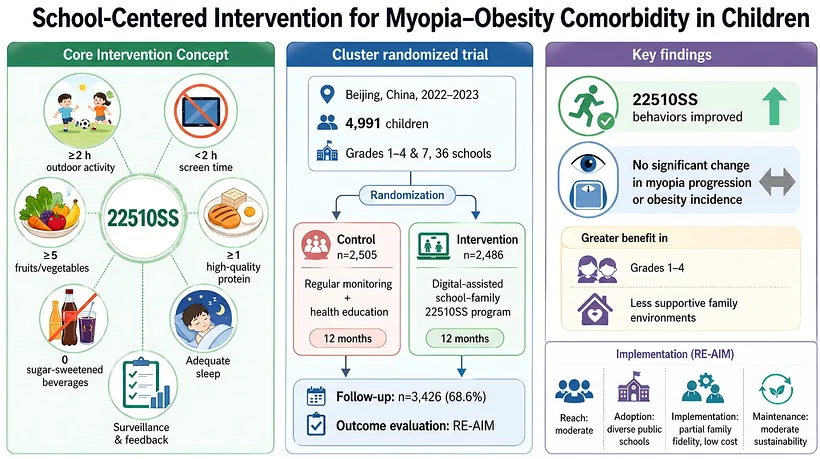

## 1 Introduction

The concurrent rise in pediatric myopia and overweight/obesity (OWOB) has become an important public health challenge, particularly in China [1, 2], where both conditions now affect large proportions of school‐aged children and adolescents. Myopia may increase the lifetime risk of pathological ocular complications, while childhood OWOB is associated with cardiometabolic abnormalities and long‐term chronic disease risk [3, 4]. Growing evidence also suggests that the coexistence of myopia and OWOB is not rare and may represent a meaningful multimorbidity burden rather than two isolated conditions [5, 6].

These conditions share several modifiable behavioral correlates, including insufficient outdoor activity, excessive sedentary screen exposure, suboptimal diet, and inadequate sleep [7, 8, 9]. Notably, increased outdoor activity has been a cornerstone strategy in interventions for both myopia and OWOB: cluster‐randomized trials and systematic reviews have shown that school‐based programs increasing outdoor time can slow myopia onset and progression in children [10, 11, 12, 13], while increased outdoor and physical activity has long been a central component of school‐based cluster‐randomized obesity‐prevention trials [14, 15, 16]. In addition, school‐based health education and family‐supported lifestyle modification have demonstrated benefits for myopia prevention or obesity prevention when delivered separately [13, 14, 17]. However, most existing programs still target a single condition, whereas children in real‐world settings are simultaneously exposed to overlapping visual, behavioral, and metabolic risks—making integrated prevention strategies that leverage these shared behavioral pathways both timely and necessary.

Integrated prevention is therefore conceptually attractive, but evidence remains limited. In particular, the “22510SS” framework was designed to address common behavioral pathways through six operational behavioral targets: at least 2 h/day of outdoor activity, no more than 2 h/day of screen time, healthy eating based on the “5/1” recommendation, zero sugar‐sweetened beverages, adequate sleep, and regular monitoring/feedback [18, 19]. Detailed intervention components and health‐education topics are provided in Tables S1 and S2. Yet demonstrating effectiveness alone is insufficient for school health policy. Whether such a program can be delivered with adequate reach, adoption, fidelity, cost control, and sustainability is equally important for multimorbidity prevention.

We therefore conducted a cluster‐randomized, hybrid type I effectiveness‐implementation trial to evaluate both the clinical effectiveness and the real‐world implementation of a 12‐month, school‐centered “22510SS” intervention for the integrated prevention of myopia and OWOB among children in routine Beijing schools. We selected the Reach, Effectiveness, Adoption, Implementation, and Maintenance (RE‐AIM) framework because it captures not only effectiveness but also population reach, organizational adoption, implementation fidelity and cost, and post‐project sustainability, all of which are critical for myopia‐OWOB comorbidity prevention, where a theoretically sound intervention may still fail to produce population benefits if family uptake, delivery intensity, and sustainability are inadequate. By simultaneously targeting shared behavioral pathways rather than any single condition, this study addresses a critical evidence gap in pediatric multimorbidity prevention and informs how integrated school‐based programs can be designed, delivered, and scaled in real‐world educational settings.

## 2 Results

### 2.1 Baseline Characteristics

The study design and participant flow are shown in Figure 1. Among 4991 enrolled children, 3426 (68.6%) completed the 12‐month follow‐up and constituted the effectiveness‐analysis sample. Participant flow and reasons for loss to follow‐up are shown in Figure S1. Attrition rates varied by grade and treatment group: completion was lowest in Grade 1 and highest in Grade 7, and participants lost to follow‐up were younger and had a lower baseline BMI than completers, suggesting potential attrition‐related selection. Baseline demographic characteristics were broadly similar between groups with respect to sex, district, single‐child status, family history of myopia or obesity, and family health environment, whereas age, school stage, and socioeconomic status differed modestly between groups (Table 1).

**FIGURE 1:**
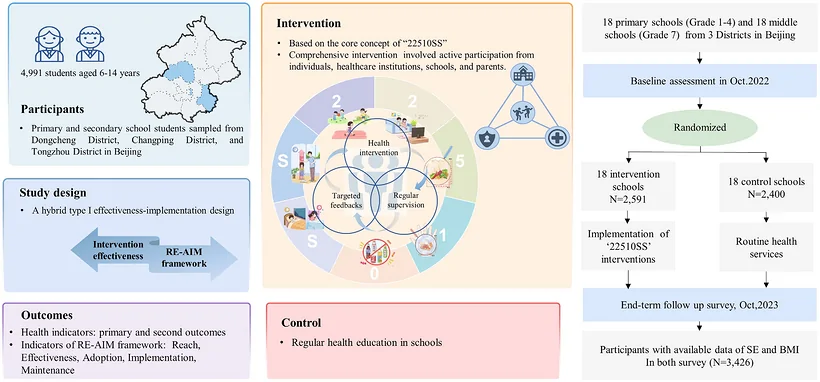
Study design of the cluster‐randomized, hybrid type I effectiveness‐implementation trial. Thirty‐six public schools in three Beijing districts (Dongcheng, Tongzhou, Changping) were pair‐matched by district and school type and randomized 1:1 to the intervention or control group. The intervention group received the 12‐month “22510SS” package (health education, environmental modification, and family/clinic‐linked monitoring), while the control group received routine school health services. Effectiveness outcomes were assessed at baseline (October 2022) and 12‐month follow‐up (October 2023); implementation outcomes were evaluated in parallel using the RE‐AIM framework. RE‐AIM, Reach, Effectiveness, Adoption, Implementation, and Maintenance.

**TABLE 1:** Baseline characteristics of participants and key baseline clinical indicators.

Characteristic	Control group (*n* = 1704)	Intervention group (*n* = 1722)	*p* value
**Sex, *n* (%)**			0.706
Boys	862 (50.6)	859 (49.9)	
Girls	842 (49.4)	863 (50.1)	
**Grade at baseline, *n* (%)**			< 0.001
Grade 1	318 (18.7)	295 (17.1)	
Grade 2	398 (23.4)	326 (18.9)	
Grade 3	379 (22.2)	363 (21.1)	
Grade 4	319 (18.7)	332 (19.3)	
Grade 7	290 (17.0)	406 (23.6)	
**Age at baseline, median [P25, P75], years**	8.70 [7.76, 10.05]	9.09 [7.92, 10.50]	< 0.001
**District, *n* (%)**			0.340
Dongcheng	577 (33.9)	564 (32.8)	
Tongzhou	560 (32.9)	544 (31.6)	
Changping	567 (33.3)	614 (35.7)	
**Single‐child status, *n* (%)**			0.427
No	783 (46.0)	767 (44.5)	
Yes	921 (54.0)	955 (55.5)	
**Socioeconomic status, *n* (%)**			< 0.001
Lower	156 (9.2)	223 (13.0)	
Moderate	1138 (66.8)	1053 (61.1)	
Upper	410 (24.1)	446 (25.9)	
**Family history of obesity, *n* (%)**			0.280
No	1255 (73.7)	1297 (75.3)	
Yes	449 (26.3)	425 (24.7)	
**Family history of myopia, *n* (%)**			0.944
No	402 (23.6)	409 (23.8)	
Yes	1302 (76.4)	1313 (76.2)	
**Family health environment, *n* (%)**			0.468
Less supportive	922 (54.1)	954 (55.4)	
Supportive	782 (45.9)	768 (44.6)	
**Baseline SE, mean ± SD, D**	−0.93 ± 1.62	−1.08 ± 1.70	0.030
**Baseline myopia prevalence, *n* (%)**	912 (53.5)	964 (56.0)	0.158
**Baseline BMI, mean ± SD, kg/m^2^**	18.58 ± 3.94	18.85 ± 4.15	0.002
**Baseline OWOB prevalence, *n* (%)**	691 (40.6)	718 (41.7)	0.518
**Baseline comorbidity prevalence, *n* (%)**	366 (21.5)	418 (24.3)	0.057

*Note*: Data are presented as mean ± SD for continuous variables and *n* (%) for categorical variables. *p* values were calculated using mixed‐effects models with schools as random effects (continuous variables: linear mixed models; categorical variables: generalized linear mixed models with logit link). Baseline SE, baseline myopia prevalence, baseline BMI, baseline OWOB prevalence, and baseline comorbidity prevalence were added to facilitate assessment of clinical comparability between groups. All adjusted models included age and grade to account for the baseline imbalance in school stage.

Abbreviations: BMI, body mass index; OWOB, overweight/obesity; SD, standard deviation; SE, spherical equivalent.

To improve assessment of baseline clinical comparability, we additionally summarize baseline spherical equivalent (SE), baseline myopia prevalence, baseline body mass index (BMI), and baseline OWOB prevalence in Table 1. Baseline myopia prevalence and OWOB prevalence were generally comparable between groups, whereas mean baseline SE and BMI were slightly less favorable in the intervention group. Because Grade 7 students were more common in the intervention group, age and grade were included as covariates in adjusted mixed‐effects models.

### 2.2 Primary and Secondary Effectiveness Outcomes

This analysis aimed to compare 12‐month changes in behavioral, visual, anthropometric, and comorbidity‐related outcomes between the intervention and control groups, and thereby to assess the overall clinical effectiveness of the “22510SS” program.

Continuous outcomes (SE and BMI) were analyzed using mixed‐effects difference‐in‐differences models, and binary outcomes (prevalence and incidence of myopia, OWOB, and their comorbidity; behavior achievement) were analyzed using mixed‐effects logistic regression, with schools treated as random effects and with adjustment for age, sex, grade, single‐child status, family history, socioeconomic status, family health environment, and baseline values, as appropriate.

Figure 2 summarizes baseline‐to‐follow‐up changes for the overall behavior‐improvement score and each primary clinical outcome, with intervention and control groups displayed side by side. Detailed results for blood glucose and lipid outcomes are provided in Table S3, and results for the individual “22510SS” behavioral indicators are shown in Figure S2. The intervention significantly improved the overall “22510SS” behavior‐improvement rate relative to the control group (39.7% vs. 33.7%; OR, 1.34; 95% CI, 1.00–1.80), indicating better uptake of the targeted health behaviors. However, no statistically significant between‐group differences were observed for any of the primary clinical outcomes: the adjusted between‐group difference in change in SE was −0.06 D (95% CI, −0.23 to 0.09); in myopia prevalence,2.1percentage points (95% CI, −2.4 to 6.9); in BMI, 0.06 kg/m^2^ (95% CI, −0.29 to 0.40); in OWOB prevalence, −0.2 percentage points (95% CI, −4.8 to 4.5); and in comorbidity prevalence, ‐0.1 percentage points (95% CI, ‐4.1 to 3.9) For secondary disease‐related outcomes, no statistically significant between‐group differences were observed in the incidence of myopia, OWOB, or comorbidity, in myopia progression, or in progression from overweight to obesity. Between‐group differences in the examined metabolic markers, including fasting glucose and triglycerides, were also not statistically significant.

**FIGURE 2:**
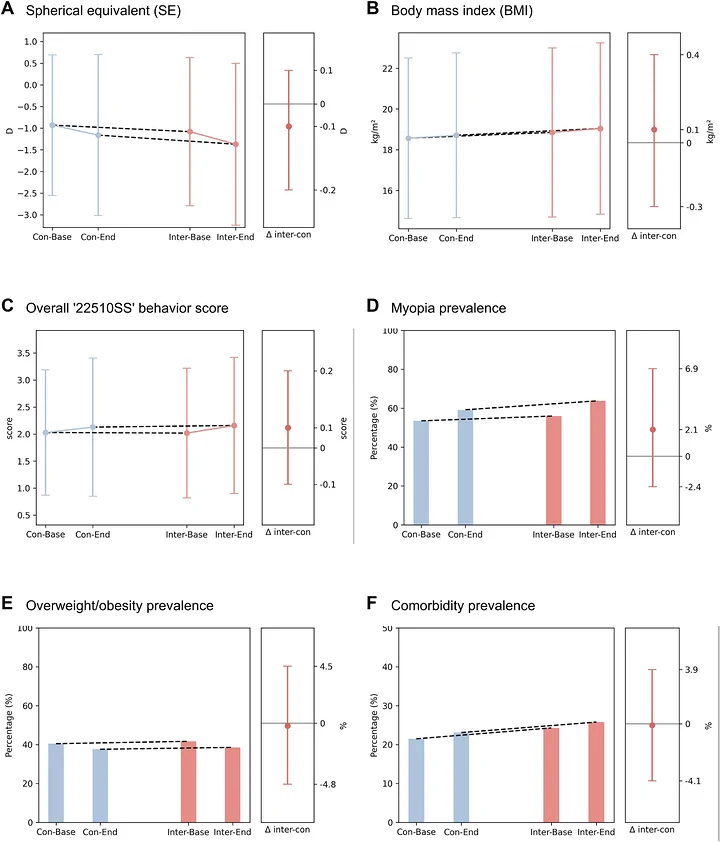
Changes from baseline to 12 months in the overall behavior‐improvement score and primary clinical outcomes. Panels show (A) spherical equivalent (SE), (B) body mass index (BMI), (C) overall “22510SS” behavior score, (D) myopia prevalence, (E) overweight/obesity (OWOB) prevalence, and (F) comorbidity prevalence. Error bars represent standard deviations for continuous outcomes and 95% confidence intervals for between‐group differences.Between‐group differences were estimated from mixed‐effects difference‐in‐differences models (continuous outcomes) or mixed‐effects logistic regression (binary outcomes), with schools as random effects and with adjustment for age, sex, grade, single‐child status, family history, socioeconomic status, family health environment, and baseline values, as appropriate. The intervention significantly improved the overall behavior‐improvement rate but produced no statistically significant differences in any of the primary clinical outcomes over 12 months. BMI, body mass index; OWOB, overweight/obesity; SE, spherical equivalent.

Overall, the 12‐month “22510SS” intervention produced measurable improvements in the targeted health behaviors but did not translate into statistically significant differences in refractive, anthropometric, or comorbidity‐related clinical outcomes over the same observation period.

### 2.3 Implementation and Safety Outcomes

The implementation analysis aimed to determine whether the limited clinical effects reflected intervention inefficacy, incomplete delivery, or both, and to characterize the program's reach, adoption, fidelity, cost, and sustainability in routine school settings.

Implementation outcomes were evaluated under the RE‐AIM framework, covering reach (participation and representativeness), effectiveness, adoption (school and staff participation), implementation (delivery fidelity, content adjustments, and costs), and maintenance (post‐trial sustainability assessed with the Program Sustainability Assessment Tool [PSAT]). Safety outcomes were summarized descriptively and compared between groups.

Figure 3 visualizes the RE‐AIM evaluation across its five dimensions, highlighting the contrast between high school‐level fidelity and lower family‐level digital engagement; Table 2 provides the corresponding quantitative indicators for each dimension. Adverse‐event outcomes are summarized in Table S4, with detailed implementation and cost results provided in Supporting Information Appendix C.

**FIGURE 3:**
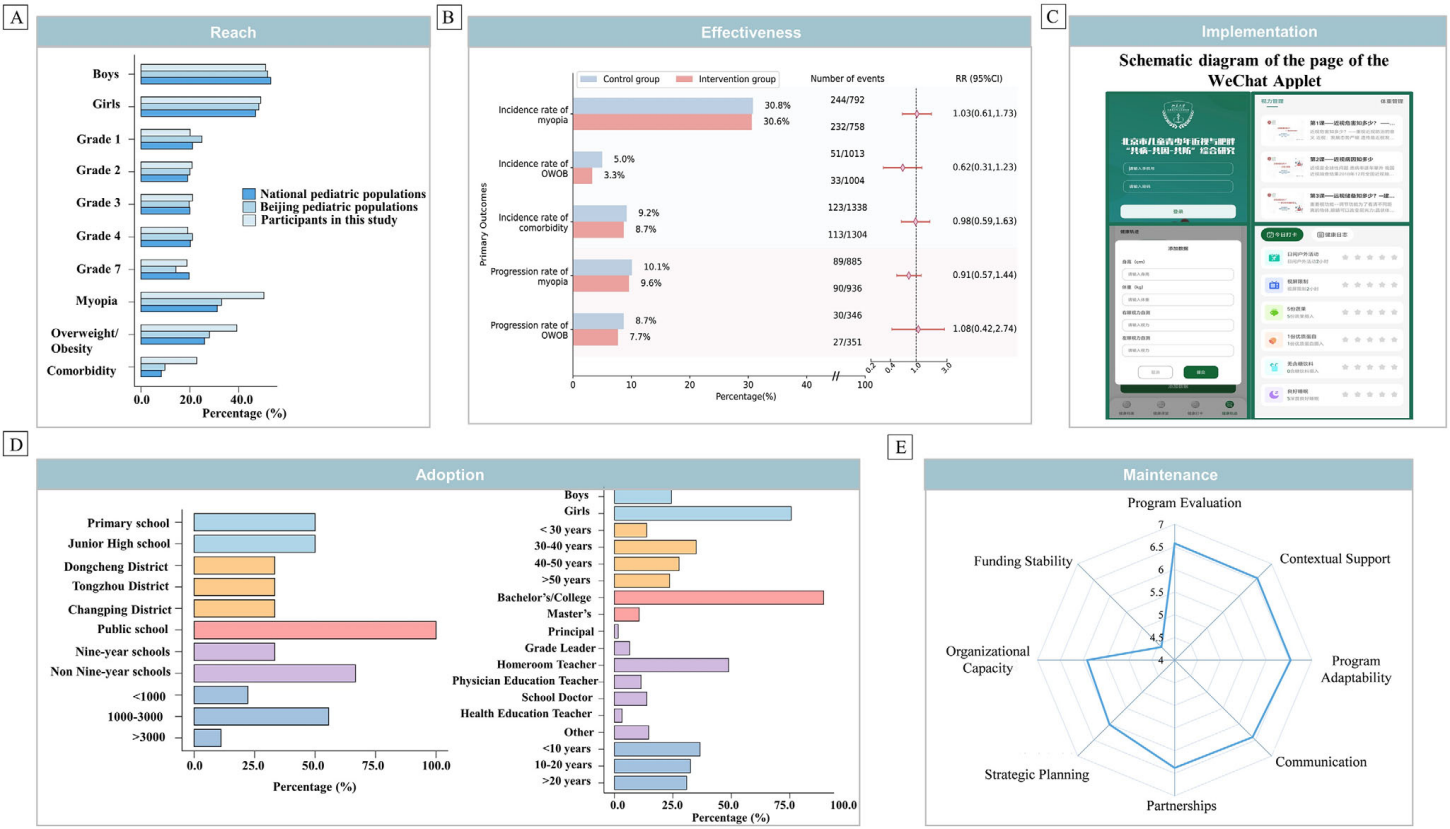
Implementation outcomes evaluated under the RE‐AIM framework. The five panels correspond to Reach (participation and representativeness), Effectiveness (behavioral and clinical outcomes), Adoption (school‐ and staff‐level participation), Implementation (delivery fidelity, content adjustments, and costs), and Maintenance (post‐trial sustainability assessed by the Program Sustainability Assessment Tool [PSAT], 1–7 scale). Quantitative indicators for each dimension are reported in Table 2. School‐level fidelity and adoption were high (100% of selected schools participated; school‐based lectures and environmental modifications were delivered with 100% fidelity), whereas family‐level engagement and digital uptake were more limited (∼60% completion of weekly health‐tip dissemination; only 594 of 2591 intervention‐group participants used the WeChat lifestyle‐monitoring applet). PSAT, Program Sustainability Assessment Tool; RE‐AIM, Reach, Effectiveness, Adoption, Implementation, and Maintenance.

**TABLE 2:** Summary of RE‐AIM implementation outcomes.

Dimension	Key metric	Result
Reach	Enrollment and representativeness	A total of 4991 students were enrolled, representing 2.6% of eligible students in the sampled grades across the three participating districts. Participant sex distribution broadly resembled regional benchmarks, but school stage and baseline health status differed from some reference populations.
Effectiveness	Primary outcomes	No statistically significant between‐group differences were observed in 12‐month changes in SE, BMI, myopia prevalence, OWOB prevalence, or comorbidity prevalence.
Effectiveness	Behavioral outcomes	The intervention improved the overall behavior‐improvement rate (39.7% vs. 33.7%; OR, 1.34; 95% CI, 1.00–1.80), but most disease‐incidence and progression outcomes remained nonsignificant overall.
Adoption	School and staff participation	All 36 selected schools participated. Implementation involved 123 school‐based staff, predominantly teachers and school health personnel.
Implementation	Fidelity	School‐based lectures and environmental modifications achieved 100% completion, whereas the family‐facing health‐tip component achieved approximately 60% completion and digital self‐monitoring uptake was limited.
Implementation	Cost	Total project cost was 998,000 yuan; per‐capita overall cost was 199.96 yuan; intervention‐group cost was 303.55 yuan/person; control‐group cost was 88.13 yuan/person; incremental per‐capita cost was 215.43 yuan. The descriptive cost was 430.85 yuan per 1 percentage point absolute difference in incident comorbidity.
Maintenance	Sustainability	The mean PSAT score was 6.10 on a 1–7 scale, with the highest score for program evaluation and the lowest score for funding stability, indicating moderate sustainability with funding as the key bottleneck.

*Note*: The RE‐AIM framework was used to evaluate program implementation across five dimensions: Reach, Effectiveness, Adoption, Implementation, and Maintenance. Reach refers to participation rate and representativeness of the target population; Effectiveness refers to behavioral and clinical outcomes; Adoption refers to school‐ and staff‐level participation; Implementation refers to delivery fidelity, content adjustments, and costs; Maintenance refers to post‐trial sustainability assessed using the Program Sustainability Assessment Tool (PSAT, 1–7 scale, with higher scores indicating greater sustainability potential). Costs are reported in Chinese yuan (¥).

Abbreviations: OWOB, overweight/obesity; PSAT, Program Sustainability Assessment Tool; RE‐AIM, Reach‐Effectiveness‐Adoption‐Implementation‐Maintenance.

The program reached 4991 students, corresponding to 2.6% of eligible students in the sampled grades across the three participating districts. School‐level adoption and delivery were strong: all 36 selected schools participated, and school‐based lectures and environmental modifications were implemented with 100% fidelity. By contrast, family‐facing components were less consistently delivered, with approximately 60% completion for weekly health‐tip dissemination and low uptake of digital self‐monitoring; only 594 participants used the lifestyle‐monitoring function of the WeChat applet, generating 11,037 behavior records, and parent engagement appeared to vary with school‐level organizational support. The total project cost was 998,000 yuan, corresponding to 199.96 yuan per participant overall; costs were calculated based on the number of participants randomized at baseline. The intervention‐group cost was 303.55 yuan per participant, the control‐group cost was 88.13 yuan per participant, and the incremental per‐participant cost was 215.43 yuan, corresponding to a descriptive cost of 430.85 yuan per 1 percentage point absolute difference in incident comorbidity. Maintenance analysis using the PSAT yielded a mean score of 6.10 on a 1–7 scale, indicating moderate sustainability potential, with program evaluation scoring highest and funding stability scoring lowest. No intervention‐related adverse events were observed, and no statistically significant between‐group differences were found for new‐onset underweight or bullying related to myopia or obesity.

In summary, the “22510SS” intervention functioned more strongly as a school‐centered than as a fully integrated school‐family program: school‐level adoption and fidelity were excellent and the incremental cost was modest, but family‐level engagement and digital uptake were the principal implementation bottlenecks, and long‐term continuation will likely depend on integration into routine school‐health budgets rather than temporary project funding.

### 2.4 Subgroup Analyses

The subgroup analyses assessed whether intervention effect estimates varied across prespecified population strata and an exploratory family‐environment stratum. Prespecified subgroup analyses were conducted by sex and school stage (grades 1–4 vs. grade 7), and a post hoc exploratory subgroup analysis examined children from more versus less supportive family health environments. Subgroup effects were estimated using the same mixed‐effects logistic regression models as the primary analysis, applied within each stratum, with schools as random effects.

Figure 4 presents a forest plot of adjusted odds ratios and 95% CI for incidence, progression, and behavior‐improvement outcomes within each subgroup. Detailed subgroup estimates for primary outcomes, secondary outcomes, and individual behavioral indicators are provided in Tables S5–S7, respectively.

**FIGURE 4:**
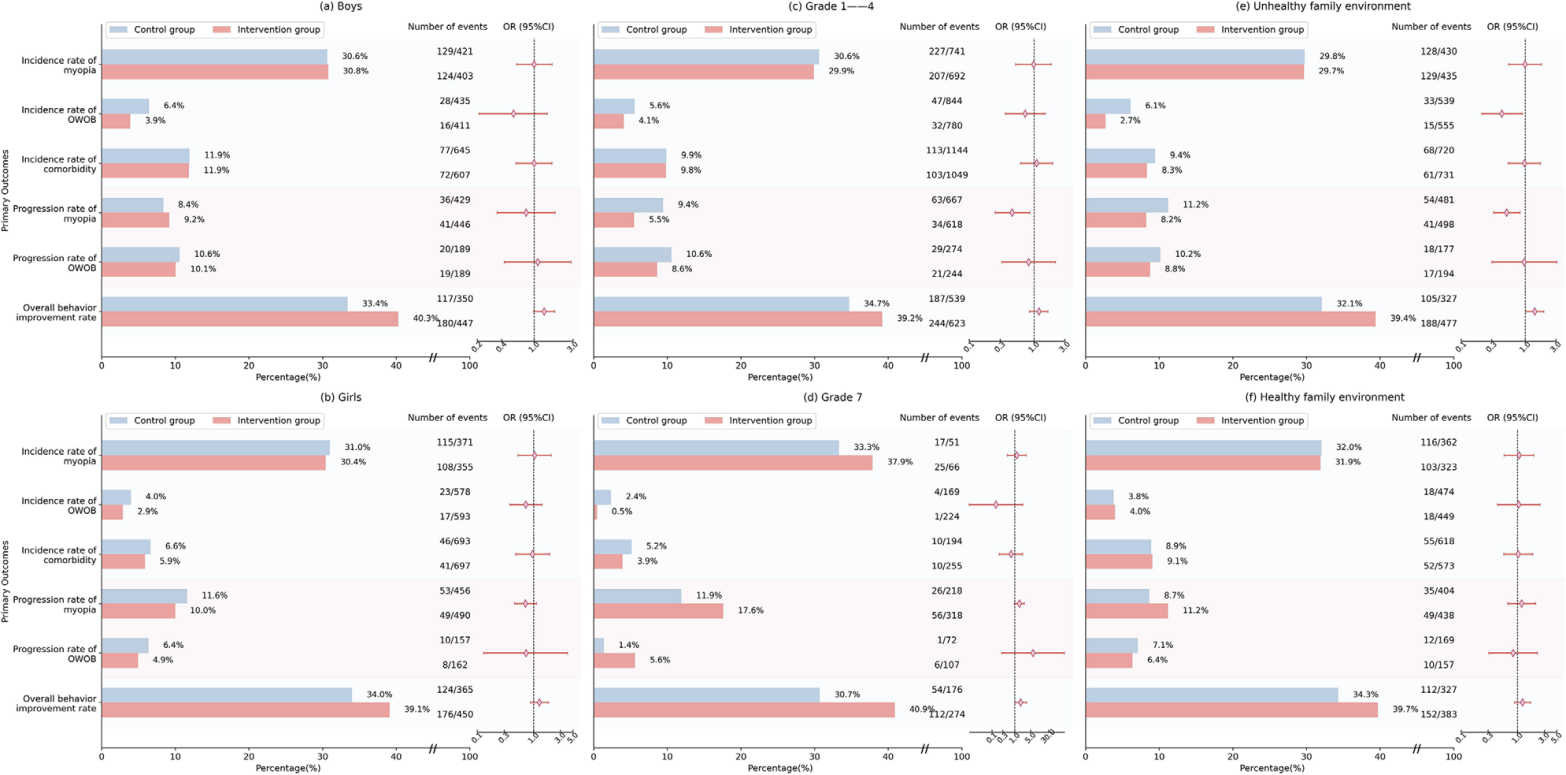
Subgroup analyses of incidence, progression, and behavior‐improvement outcomes over 12 months. Forest plot shows adjusted ORs and 95% CIs for each subgroup, estimated from mixed‐effects logistic regression models with schools as random effects. Prespecified subgroup analyses were conducted for sex and school stage; the family‐health‐environment subgroup analysis was exploratory. For incidence and progression outcomes, an OR below 1 favors the intervention; for behavior improvement, an OR above 1 favors the intervention. Some subgroup estimates were statistically significant, but these findings should be interpreted cautiously because the primary outcomes were null and multiple subgroup comparisons were conducted. The post hoc family‐environment findings are hypothesis‐generating only and require confirmation in future studies. CI, confidence interval; OR, odds ratio; OWOB, overweight/obesity.

Most subgroup estimates were not statistically significant. In the prespecified school‐stage analysis, students in Grades 1–4 in the intervention group showed significantly slower myopia progression than their counterparts in the control group (OR, 0.46; 95% CI, 0.25–0.86), whereas no corresponding effect was observed in Grade 7. No significant differences were observed in the prespecified sex‐stratified analysis. In the exploratory family‐environment analysis, children from less supportive family health environments showed lower OWOB incidence (OR, 0.43; 95% CI, 0.21–0.90), slower myopia progression (OR, 0.51; 95% CI, 0.32–0.83), and greater overall behavior improvement (OR, 1.41; 95% CI, 1.02–1.95) in the intervention group than in the control group.

These subgroup estimates should not be interpreted as evidence of subgroup‐specific efficacy. Because the primary outcomes were null, only sex and school stage were prespecified, the family‐environment analysis was post hoc, and multiple subgroup comparisons were conducted, the family‐environment findings are hypothesis‐generating only and require confirmation in future adequately powered studies with prespecified subgroup hypotheses.

## 3 Discussion

This cluster‐randomized trial evaluated the effectiveness and implementation of the “22510SS” intervention for the co‐prevention of myopia and OWOB in school‐aged children. The main finding was that the intervention improved targeted behaviors, but these gains did not translate into statistically significant population‐level improvements in the primary clinical outcomes over 12 months. At the same time, prespecified analyses suggested slower myopic progression in Grades 1–4, and exploratory analyses suggested additional benefits among children from less supportive family health environments.

These findings should be interpreted in the context of prior school‐based prevention trials, most of which have targeted myopia or obesity in isolation. Several large cluster‐randomized trials of school‐based obesity prevention, including the WAVES trial in 6‐ to 7‐year‐old UK children, the HeLP trial in UK primary schools, and the CHIRPY DRAGON trial in Chinese primary schools, similarly reported no significant between‐group differences in BMI or adiposity at 12–24 months, despite favorable changes in targeted behaviors [15, 16, 20]. A more recent Chinese cluster‐randomized trial of a multifaceted obesity‐prevention program in primary‐school children also produced only modest anthropometric effects despite high implementation fidelity [14, 21]. On the myopia side, network meta‐analyses and systematic reviews have shown that meaningful slowing of refractive progression in school‐based settings typically requires sustained, well‐supervised increases in outdoor time or pharmacological/optical interventions, with behavioral‐only programs generally yielding small effects over 12 months [12, 13, 22]. Our results extend this literature in two ways. First, we show that an integrated, multi‐behavioral package targeting shared risk factors for myopia and OWOB is feasible to deliver in routine schools but, like most single‐disease behavioral programs, does not produce statistically significant population‐level clinical effects within 12 months. Second, by jointly evaluating implementation, we demonstrate that the null clinical findings cannot be attributed to poor school‐level delivery; rather, they appear to reflect the limited intensity, duration, and family‐level penetration that have repeatedly constrained behavioral school‐based trials in this field.

The RE‐AIM framework was particularly useful for interpreting this trial because the implementation findings help explain the limited clinical effects. School‐based delivery was strong, but the family‐facing components showed substantial attenuation. This mismatch is important because several intervention targets, including after‐school outdoor time, home screen use, sleep schedules, and dietary practices, are strongly shaped by families rather than schools alone [17, 23, 24, 25, 26]. The data therefore suggest that the intervention done outside school was likely lower than intended. This may also help explain why not all behavioral components responded equally well: reducing discretionary screen time may have been easier to reinforce through repeated messaging and monitoring than achieving more than 2 h/day of outdoor activity, which depends more heavily on time, weather, school routines, and family organization. In this sense, the trial may be viewed as demonstrating good school‐level feasibility but incomplete family‐level penetration.

The low uptake of the WeChat applet provides a concrete example. Parent participation appeared to depend heavily on how actively schoolteachers and school health staff promoted the platform, and repeated app‐based check‐ins may also have created reporting burden. In addition, the applet mainly served monitoring and feedback functions and likely lacked more engaging features that might sustain long‐term participation. For future iterations, simpler workflows, stronger teacher endorsement, automated reminders, and more motivating digital design may improve family adherence.

The subgroup findings are potentially informative for future implementation. Younger students may be more behaviorally and biologically responsive to prevention efforts before refractive and metabolic trajectories are fully established, which may partly explain the slower myopic progression observed in Grades 1–4 [27, 28]. The exploratory findings in children from less supportive family health environments are also noteworthy from a health‐equity perspective: these children may benefit most from additional structure and reinforcement provided by school‐based programs [17, 29, 30, 31]. Nevertheless, these results must be interpreted cautiously because the primary outcomes were null, only sex and school stage were prespecified subgroup analyses, and residual multiplicity remains a concern.

This trial has several strengths. It addressed an important but understudied multimorbidity problem, used a cluster‐randomized design in routine schools, combined effectiveness and implementation evaluation within a hybrid design, and generated practical evidence on reach, fidelity, cost, and sustainability. Nonetheless, the following methodological limitations should be noted. First, myopia was defined using non‐cycloplegic SE (≤ −0.50 D). This approach is feasible for large‐scale school studies but may overestimate myopia prevalence, particularly in younger children because of accommodative effects. The resulting bias would most likely shift SE in the myopic direction and should be interpreted as an epidemiologic screening definition rather than a clinical diagnosis. Because the same protocol and equipment were used in both groups, this bias was likely nondifferential with respect to randomized allocation, but it may still have reduced precision and affected the absolute prevalence estimates. Second, the primary effectiveness analyses were based on participants with complete follow‐up outcome data (3426 of 4991 enrolled; 68.6% completion). Attrition was associated with age and baseline BMI; participants lost to follow‐up were younger and had lower baseline BMI than completers. Completion rates were broadly similar between the intervention and control groups. Nevertheless, this selective attrition may have affected the representativeness and precision of the estimates. No formal sensitivity analysis (e.g., inverse probability weighting or multiple imputation) was performed to adjust for potential attrition bias, which should be considered a limitation. Third, behavioral outcomes were self‐reported and may have been influenced by social desirability or differential reporting, potentially biasing estimates toward apparent improvement in the intervention group or, alternatively, toward the null if both groups became more aware of recommended behaviors. Fourth, because the intervention was multi‐component, we could not isolate the contribution of each component, and objective process data were stronger for some intervention elements than for others. Fifth, we excluded orthokeratology users but did not collect information on all other possible myopia‐control treatments, such as atropine or defocus spectacle lenses, which may have introduced residual confounding in refractive outcomes. Sixth, the study reached only a modest proportion of eligible students in the sampled districts, which may limit external validity even though the participating schools covered diverse urban contexts. Finally, the 12‐month duration may have been sufficient for behavioral change but still short for broader physiological effects on refractive or metabolic endpoints, as longer follow‐up is often required in school‐based vision and weight interventions.

Several future directions follow from these findings. First, future iterations of integrated multimorbidity prevention should strengthen family‐level engagement through more interactive and lower burden digital tools, structured parent‐training components, or scheduled home‐school communication, rather than relying primarily on passive information dissemination. Second, programs may achieve greater clinical impact by prioritizing younger children, in whom refractive and metabolic trajectories are still modifiable, and by allocating additional support to families with limited baseline health resources. Third, longer follow‐up of at least 24–36 months will be needed to determine whether sustained behavioral improvements eventually translate into measurable refractive and anthropometric benefits [32, 33]. Fourth, future trials should consider embedding objective process measures, such as wearable‐device‐based outdoor‐time monitoring or routine school‐health‐record linkage, to reduce reliance on self‐report and to better quantify the actual delivered dose. Finally, given the moderate PSAT sustainability score and the current dependence on temporary project funding, embedding the core “22510SS” components into routine school‐health budgets and existing district‐level health policies will be critical for long‐term sustainability and equitable scale‐up [34].

In summary, the “22510SS” cluster‐randomized trial showed that an integrated, school‐centered program for the joint prevention of myopia and OWOB was feasible to deliver with high school‐level fidelity at modest incremental cost and produced measurable improvements in targeted health behaviors. However, these behavioral gains did not translate into statistically significant population‐level improvements in refractive, anthropometric or comorbidity outcomes within 12 months, and the observed subgroup signals in younger children and those from less supportive family environments should be regarded as hypothesis‐generating rather than confirmatory and require confirmation in future prespecified studies. Together with prior school‐based prevention trials, these findings suggest that integrated multimorbidity prevention is operationally feasible, but that stronger family engagement, longer follow‐up and policy‐level embedding will be essential before behavioral change can be expected to translate into sustained clinical and public‐health benefit.

## 4 Materials and Methods

### 4.1 Trial Design and Participants

We conducted a cluster‐randomized, hybrid type I effectiveness‐implementation trial from September 2022 to October 2023 in 36 public schools across three socioeconomically diverse districts of Beijing (Dongcheng, Tongzhou, and Changping) [35, 36]. Schools were pair‐matched by district and school type and then randomized 1:1 to intervention or control. The study design and trial registration have been published previously in the study protocol.

A total of 18 primary schools and 18 junior high schools were included. Eligible participants were students in Grades 1–4 and Grade 7. These grades were selected to ensure complete 12‐month follow‐up within the same school. Students in Grades 5–6 would face the primary‐to‐junior‐high transition, and those in Grades 8–9 would approach the senior‐high entrance examination, both of which would have caused substantial loss to follow‐up. Students were excluded if they had major systemic diseases or physical conditions limiting participation in school activities, ophthalmic conditions affecting refraction assessment, a transfer plan within 1 year, or orthokeratology use.

### 4.2 Randomization and Masking

After baseline assessment, an independent statistician at the Peking University Clinical Research Institute performed central randomization using a computer‐generated sequence, stratified by district and school type. Allocation was concealed until schools had completed baseline assessment. Because of the nature of the behavioral intervention, blinding of participants and school personnel was not feasible; however, measurement staff followed standardized procedures and were not involved in intervention delivery.

### 4.3 Intervention

The 12‐month “22510SS” intervention targeted shared behavioral pathways for myopia and OWOB through a school‐centered, multicomponent strategy. The program emphasized six operational behavioral domains assessed in the effectiveness analysis: (1) at least 2 h/day of outdoor activity, (2) no more than 2 h/day of screen time, (3) healthy eating according to the combined “5/1” recommendation (≥ 5 servings of fruits and vegetables plus 1 serving of high‐quality protein daily), (4) zero sugar‐sweetened beverages, (5) age‐appropriate sleep duration, and (6) regular health monitoring and feedback.

The intervention included three major delivery components. First, health education was provided through online videos, offline classroom sessions, and live lectures for students, parents, and teachers. Second, environmental reinforcement, which was distinct from formal didactic health education, was implemented through school posters, educational pamphlets, visual prompts, and routine dissemination of evidence‐based health messages to provide continuous behavioral cues outside discrete teaching sessions. Third, behavioral monitoring and feedback were supported by a WeChat applet through which parents could report lifestyle indicators and receive individualized prompts. Higher risk students received additional monitoring, school‐nurse support, and referral to clinical partners when appropriate. The planned delivery frequency and duration of each component are detailed in Table 3. In brief, the delivered dose was highest for school‐based educational activities (12 sessions for students, 4 for parents, and 2 for teachers over 12 months, all completed as planned), moderate for environmental reinforcement (continuous display with semester updates), and dependent on family participation for digital monitoring and home‐based components.

**TABLE 3:** Intervention components, delivery intensity, and expected dose.

Component	Main delivery mode	Planned frequency and duration	Active family participation required	Expected delivery certainty
Health education	Recorded videos, in‐person classes, live lectures for students, parents, and teachers	Students: 12 sessions × 40 min over 12 months (every 2–3 weeks); parents: 4 sessions × 60 min (2 per semester); teachers: 2 sessions × 60 min (1 per semester)	Partial	High
Environmental reinforcement	Posters, pamphlets, branded materials, routine message dissemination	Continuous display throughout 12 months; materials updated each semester	Limited	High within schools
Digital monitoring and feedback	WeChat applet reporting, reminders, individualized feedback	Monthly behavioral check‐in; weekly health‐tip push notifications	Yes	Moderate to low
Targeted support for higher risk students	School‐nurse follow‐up, parent feedback, referral when appropriate	As needed, based on risk screening at baseline and mid‐point	Yes	Moderate

*Note*: The “22510SS” intervention comprised three major delivery components (health education, environmental modification, and family/clinic‐linked monitoring) targeting six operational behavioral domains. Planned frequency and duration reflect the protocol‐specified dose; actual delivery matched the planned dose for all school‐based components (100% fidelity). Family‐facing digital components had variable uptake.

### 4.4 Control Group

Children and adolescents in control schools received routine school health education and regular monitoring, but they did not receive the structured “22510SS” package, digital monitoring/feedback system, or the enhanced family‐ and clinic‐linked components. The principal contrast between groups was therefore not the presence versus absence of any health education, but the addition of a higher frequency, multi‐channel, feedback‐supported intervention in the experimental arm.

### 4.5 Assessments

Baseline assessments were conducted in October 2022 and follow‐up assessments in October 2023 by trained staff using standardized protocols. Uncorrected distance visual acuity was measured with standard logarithmic charts, and non‐cycloplegic refraction was measured using a tabletop autorefractor (Topcon KR‐800, Topcon Corporation, Tokyo, Japan). Three measurements were taken per eye, and SE was calculated as the sphere plus half of the cylinder. If any two measurements differed by at least 0.50 D, additional measurements were taken and averaged.

Anthropometric assessments included height, weight, waist circumference, hip circumference, and body fat percentage. BMI was calculated as weight/height^2^ (kg/m^2^), and OWOB was classified according to WHO standards [37]. Fasting venous blood samples were collected and analyzed for glucose, triglycerides, total cholesterol, high‐density lipoprotein cholesterol, and low‐density lipoprotein cholesterol. Questionnaires were used to collect demographic, behavioral, and family‐environment information; for younger children, parents assisted questionnaire completion to ensure comprehension.

### 4.6 Outcome Measures

Primary outcomes were the changes from baseline to 12 months in SE, BMI, and the prevalence of myopia, OWOB, and their comorbidity. Changes in SE and BMI were prespecified as primary clinical outcomes in the trial protocol [35]. Prevalence‐based outcomes were also designated as primary because they could be estimated irrespective of baseline disease status among participants with available follow‐up outcome data, thereby capturing the overall population‐level disease burden in this pragmatic trial. Myopia was defined epidemiologically as non‐cycloplegic SE ≤ −0.50 D, with severity categorized according to the International Myopia Institute classification as low (−3.00 D < SE ≤ −0.50 D), moderate (−6.00 D < SE ≤ −3.00 D), or high (SE ≤ −6.00 D) [38, 39].

Secondary disease‐related outcomes comprised incidence and progression measures, which were inherently restricted to participants who were disease‐free or met prespecified severity criteria at baseline. Incidence of myopia, OWOB, and comorbidity was defined as the proportion of participants without the relevant condition at baseline who met its definition at follow‐up. Myopia progression was defined as advancement to a more severe prespecified category (e.g., low to moderate, or moderate to high) at follow‐up, assessed only among participants with low or moderate myopia at baseline; those without myopia contributed to the incidence outcome, and those with high myopia had no more severe category to progress to. OWOB progression was defined analogously as transition from overweight to obesity at follow‐up, assessed only among participants who were overweight at baseline.

Behavior‐related secondary outcomes were the achievement rate for each of the six “22510SS” behaviors at follow‐up and the overall behavior‐improvement rate. The six behaviors were as follows: (1) ≥ 2 h/day of outdoor activity; (2) ≤ 2 h/day of screen time; (3) healthy eating (combining ≥ 5 servings of fruits/vegetables and 1 serving of high‐quality protein daily); (4) 0 sugar‐sweetened beverages; (5) adequate sleep (≥ 10 h for primary‐school students, ≥ 9 h for junior‐high students); and (6) regular health monitoring and feedback. Each behavior was scored as achieved (1) or not achieved (0), and a six‐item composite score (range, 0–6) was calculated by equally weighted summation. The overall behavior‐improvement rate was defined as an increase of at least 1 point in this composite score from baseline to follow‐up. The composite was developed by the study team as a pragmatic evaluation measure rather than a previously validated scale.

Implementation outcomes were evaluated using the RE‐AIM framework, in which reach denoted participation and representativeness; effectiveness, clinical, and behavioral outcomes; adoption, school, and staff participation; implementation, delivery fidelity, content adjustments, and costs; and maintenance, post‐project sustainability. Details of the development of the RE‐AIM‐based implementation outcome indicators are provided in Supporting Information Appendix A.

### 4.7 Adverse Events

Adverse events included intervention‐related injury incidents, new‐onset underweight, and self‐reported bullying related to myopia or obesity during follow‐up.

### 4.8 Statistical Analysis

Sample size was determined a priori for a cluster‐randomized design assuming a 1:1 allocation ratio, an intraclass correlation coefficient of 0.03, and clinically meaningful differences in SE and BMI. The final enrollment target also accounted for anticipated attrition. Effectiveness analysis was conducted among participants with available 12‐month follow‐up outcome data. Baseline differences were assessed using mixed‐effects models with schools as random effects, while attrition patterns were assessed using chi‐square tests, Mann–Whitney tests, or *t* tests as appropriate.

Continuous outcomes were analyzed using mixed‐effects difference‐in‐differences models, and binary outcomes were analyzed using mixed‐effects logistic regression models with schools treated as random effects. Fixed‐effect covariates included age, sex, grade, single‐child status, family history of myopia or obesity, socioeconomic status, and family health environment, as appropriate to the model. Missing covariate data were imputed using a random‐forest approach. The definition and coding of socioeconomic status are provided in Supporting Information Appendix B. Prespecified subgroup analyses were performed for sex and school stage, with interaction *p* values reported where applicable; the family‐health‐environment analysis was post hoc and exploratory. Family health environment was defined using four equally weighted parental indicators selected to reflect family‐level support for the core “22510SS” behavioral domains: allowing sufficient time for children's outdoor physical activity (corresponding to the outdoor‐activity target) [40, 41, 42], restricting recreational screen time (corresponding to the screen‐time target), using nutrition labels when purchasing food (corresponding to the healthy‐eating target), and awareness of the importance of regular monitoring for comorbid myopia and obesity (corresponding to the monitoring target) [40, 41]. Equal weighting was applied for consistency with the equally weighted “22510SS” composite behavior score. Participants meeting all four criteria were classified as having a supportive family health environment; otherwise, the environment was classified as less supportive. Costs were calculated using the numbers of participants randomized at baseline. The incremental per‐participant cost was calculated as the intervention‐group per‐participant cost minus the control‐group per‐participant cost. A descriptive cost per 1 percentage point absolute difference in incident comorbidity was calculated by dividing the incremental per‐participant cost by the absolute between‐group difference in incident comorbidity. All tests were two‐sided, and *p* < 0.05 indicated statistical significance. Analyses were performed using Python 3.12 and R 4.3.2.

## Author Contributions

Yi Song and Yanhui Dong conceived and designed the study and took responsibility for data integrity and the accuracy of the data analysis. Jing Li, Di Shi, Yaqi Wang, Yihang Zhang, Jiajia Dang, Yunfei Liu, Jieyu Liu, and Ziyue Chen contributed to data curation, project implementation, and formal analysis. Xinxin Wang, Jieyun Song, Jun Ma, and Randall S. Stafford contributed to methodology development, interpretation of findings, and critical revision of the manuscript. Jing Li, Di Shi, and Yaqi Wang drafted the manuscript. Yi Song and Jing Li verified the underlying data. All authors had full access to the data, had final responsibility for the decision to submit, and have read and approved the final manuscript.

## Funding

The present study was funded by the National Key Research and Development Program of China (Grant No. 2024YFC2707901 to Yi Song and 2024YFC3308302 to Yanhui Dong) and National Natural Science Foundation of China (Grant No. 82273654 to Yi Song, 82373593 and 82103865 to Yanhui Dong).

## Ethics Statement

This study was approved by the Peking University Health Science Center Ethics Review Board (IRB00001052‐22018). Informed consent was obtained from all participants and their legal guardians.

## Conflicts of Interest

The authors declare no conflicts of interest.

## Supporting information




**Supporting Information**: mco271092‐sup‐0001‐SupMat.docx

## Data Availability

Due to ethical restrictions on minor participant data imposed by the Peking University Health Science Center Ethics Review Board, the deidentified data are not publicly available. Qualified researchers may request access from the corresponding author 24 months after publication, subject to an approved proposal and a signed data sharing agreement.

## References

[1] P. N. Baird , S. M. Saw , C. Lanca , et al., “Myopia,” Nature Reviews Disease Primers 6, no. 1 (2020): 99. 10.1038/s41572-020-00231-4 33328468

[2] World Obesity Federation, "World Obesity Atlas 2023," World Obesity Federation, published March 2023, https://data.worldobesity.org/publications/?cat=19.

[3] P. L. Zhong , N. Ma , Y. F. Liu , et al., “[Trend of the Detection Rate of Myopia Among Chinese Han Children and Adolescents Aged 7–18 Years From 2010 to 2019],” Zhonghua Yu Fang Yi Xue Za Zhi [Chinese Journal of Preventive Medicine] 57 (2023): 20–26. 10.3760/cma.j.cn112150-20221008-00964 36854441

[4] Y. H. Dong , L. Chen , J. Y. Liu , et al., “Epidemiology and Prediction of Overweight and Obesity Among Children and Adolescents Aged 7–18 Years in China From 1985 to 2019],” Zhonghua Yu Fang Yi Xue Za Zhi [Chinese Journal of Preventive Medicine] 57 (2023): 11–19. 10.3760/cma.j.cn112150-20220906-00881 36854438

[5] J. Dang , S. Cai , P. Zhong , et al., “Associations of School Physical Activity Environment With Comorbid Obesity and Myopia in Children and Adolescents: Findings From a Chinese National Follow‐Up Study,” Scandinavian Journal of Medicine & Science in Sports 34, no. 1 (2024): e14562. 10.1111/sms.14562 38268065

[6] C. Yin , Q. Gan , P. Xu , et al., “Weight Status and Myopia in Children and Adolescents: A Nationwide Cross‐Sectional Study of China,” Nutrients 17, no. 2 (2025): 260. 10.3390/nu17020260 PMC11767739 39861390

[7] J. Mu , H. Zhong , and M. Jiang , “Risk Factors and Cumulative Risk of Comorbid Myopia and Obesity Among Children and Adolescents in Southern China: A Cross‐sectional Study,” Health Sci Reports 8, no. 7 (2025): e71087. 10.1002/hsr2.71087 PMC12272303 40687543

[8] S. Biswas , A. El Kareh , M. Qureshi , et al., “The Influence of the Environment and Lifestyle on Myopia,” Journal of Physiological Anthropology 43, no. 1 (2024): 7. 10.1186/s40101-024-00354-7 PMC10829372 38297353

[9] Y. Dong , C. Yuan , J. Dang , et al., “Control of Childhood Obesity and Implications for Policy in China,” Lancet Public Health 9, no. 12 (2024): e1125–e1135. 10.1016/S2468-2667(24)00263-9 39579776

[10] Y. Qu , J. Yu , W. Xia , and H. Cai , “Correlation of Myopia With Physical Exercise and Sleep Habits Among Suburban Adolescents,” Journal of Ophthalmology 2020 (2020): 2670153. 10.1155/2020/2670153 PMC7399760 32774900

[11] L. Cordain , S. B. Eaton , J. Brand Miller , S. Lindeberg , and C. Jensen , “An Evolutionary Analysis of the Aetiology and Pathogenesis of Juvenile‐Onset Myopia,” Acta Ophthalmologica Scandinavica 80, no. 2 (2002): 125–135. 10.1034/j.1600-0420.2002.800203.x 11952477

[12] J. G. Lawrenson , R. Shah , B. Huntjens , et al., “Interventions for Myopia Control in Children: A Living Systematic Review and Network Meta‐Analysis,” Cochrane Database of Systematic Reviews 2, no. 2 (2023): CD014758. 10.1002/14651858.CD014758.pub2 PMC9933422 36809645

[13] M. He , F. Xiang , Y. Zeng , et al., “Effect of Time Spent Outdoors at School on the Development of Myopia Among Children in China: A Randomized Clinical Trial,” JAMA 314, no. 11 (2015): 1142–1148. 10.1001/jama.2015.10803 26372583

[14] Z. Liu , P. Gao , A. Y. Gao , et al., “Effectiveness of a Multifaceted Intervention for Prevention of Obesity in Primary School Children in China: A Cluster Randomized Clinical Trial,” JAMA Pediatrics 176, no. 1 (2022): e214375. 10.1001/jamapediatrics.2021.4375 PMC8576631 34747972

[15] J. Lloyd , S. Creanor , S. Logan , et al., “Effectiveness of the Healthy Lifestyles Programme (HeLP) to Prevent Obesity in UK Primary‐School Children: A Cluster Randomised Controlled Trial,” Lancet Child & Adolescent Health 2, no. 1 (2018): 35–45. 10.1016/S2352-4642(17)30151-7 PMC5738974 29302609

[16] B. Li , M. Pallan , W. J. Liu , et al., “The CHIRPY DRAGON Intervention in Preventing Obesity in Chinese Primary‐School‐Aged Children: A Cluster‐Randomised Controlled Trial,” Plos Medicine 16, no. 11 (2019): e1002971. 10.1371/journal.pmed.1002971 PMC6879117 31770371

[17] F. Santos , H. Sousa , E. R. Gouveia , et al., “School‐Based family‐Oriented Health Interventions to Promote Physical Activity in Children and Adolescents: A Systematic Review,” American Journal of Health Promotion 37, no. 2 (2023): 243–262. 10.1177/08901171221113836 PMC9850376 36413351

[18] A. S. Khalsa , R. Kharofa , N. J. Ollberding , L. Bishop , and K. A. Copeland , “Attainment of “5‐2‐1‐0” Obesity Recommendations in Preschool‐Aged Children,” Preventive Medicine Reports 8 (2017): 79–87. 10.1016/j.pmedr.2017.08.003 PMC5573793 28856085

[19] V. W. Rogers and E. Motyka , “5‐2‐1‐0 Goes to School: A Pilot Project Testing the Feasibility of Schools Adopting and Delivering Healthy Messages During the School Day,” Pediatrics 123, no. S5 (2009): S272–S276. 10.1542/peds.2008-2780E 19470603

[20] P. Adab , M. J. Pallan , E. R. Lancashire , et al., “Effectiveness of a Childhood Obesity Prevention Programme Delivered Through Schools, Targeting 6‐ and 7‐Year‐Olds: Cluster Randomised Controlled Trial (WAVES Study),” Bmj 360 (2018): k211. 10.1136/bmj.k211 PMC5792961 29437667

[21] P. Guo , Y. Zhou , Z. Zou , et al., “Effects of School‐Based Lifestyle Interventions on Cardiovascular Health in Chinese Children and Adolescents: A Post Hoc Analysis of a National Multicenter Study,” Journal of the American Heart Association 14, no. 1 (2025): e037371. 10.1161/JAHA.124.037371 PMC12054418 39704211

[22] C. F. Wildsoet , A. Chia , P. Cho , et al., “IMI—Interventions Myopia Institute: Interventions for Controlling Myopia Onset and Progression Report,” Investigative Ophthalmology & Visual Science 60, no. 3 (2019): M106–M131. 10.1167/iovs.18-25958 30817829

[23] M. C. A. Bontje , R. W. de Ronde , E. M. Dubbeldeman , M. Kamphuis , R. Reis , and M. R. Crone , “Parental Engagement in Preventive Youth Health Care: Effect Evaluation,” Children and Youth Services Review 120 (2021): 105724.

[24] N. Axford , M. Lehtonen , D. Kaoukji , K. Tobin , and V. Berry , “Engaging Parents in Parenting Programs: Lessons From Research and Practice,” Children and Youth Services Review 34, no. 10 (2012): 2061–2071.

[25] S. Park , S. I. Stone , and S. D. Holloway , “School‐Based Parental Involvement as a Predictor of Achievement and School Learning Environment: An Elementary School‐Level Analysis,” Children and Youth Services Review 82 (2017): 195–206.

[26] L. Xiong , “Challenges and Facilitators to Parent‐Child Shared Physical Interventions During Preschool and School Age Education: A Systematic Review and Meta Synthesis,” Front Public Health 13 (2025): 1658179. 10.3389/fpubh.2025.1658179 PMC12627017 41268416

[27] J. J. Walline , K. B. Lindsley , S. S. Vedula , et al., “Interventions to Slow Progression of Myopia in Children,” Cochrane Database of Systematic Reviews 1, no. 1 (2020): CD004916. 10.1002/14651858.CD004916.pub4 PMC6984636 31930781

[28] D. O. Mutti , J. R. Hayes , G. L. Mitchell , et al., “Refractive Error, Axial Length, and Relative Peripheral Refractive Error Before and After the Onset of Myopia,” Investigative Ophthalmology & Visual Science 48, no. 6 (2007): 2510–2519. 10.1167/iovs.06-0562 PMC2657719 17525178

[29] Y. Demetriou and O. Höner , “Physical Activity Interventions in the School Setting: A Systematic Review,” Psychology of Sport and Exercise 13, no. 2 (2012): 186–196.

[30] E. Haataja , H. Leppä , M. Huhtiniemi , et al., “Social Inequalities in the Effects of School‐Based Well‐Being Interventions: A Systematic Review,” European Journal of Public Health 35, no. 2 (2025): 302–311. 10.1093/eurpub/ckaf005 PMC11967906 39977576

[31] L. R. Glasman and D. Albarracín , “Forming Attitudes That Predict Future Behavior: A Meta‐Analysis of the Attitude‐Behavior Relation,” Psychological Bulletin 132, no. 5 (2006): 778–822. 10.1037/0033-2909.132.5.778 PMC4815429 16910754

[32] E. Shneor , L. A. Ostrin , A. Gordon‐Shaag , et al., “Twelve Month Refractive and Axial Length Changes in the Israeli Refractive Error, Activity, and Devices (iREAD) Study,” Scientific Reports 15, no. 1 (2025): 12778. 10.1038/s41598-025-97336-1 PMC11997210 40229333

[33] D. B. Cunha , S. N. de Souza Bda , R. A. Pereira , and R. Sichieri , “Effectiveness of a Randomized School‐Based Intervention Involving Families and Teachers to Prevent Excessive Weight Gain Among Adolescents in Brazil,” PLoS ONE 8, no. 2 (2013): e57498. 10.1371/journal.pone.0057498 PMC3581462 23451237

[34] S. Stoll , M. Janevic , M. Lara , et al., “A Mixed‐Method Application of the Program Sustainability Assessment Tool to Evaluate the Sustainability of 4 Pediatric Asthma Care Coordination Programs,” Preventing Chronic Disease 12 (2015): E214. 10.5888/pcd12.150133 PMC4674447 26632955

[35] X. Wang , J. Dang , J. Liu , et al., “A Cluster Randomized Trial of a Comprehensive Intervention Nesting Family and Clinic Into School‐Centered Implementation to Reduce Myopia and Obesity Among Children and Adolescents in Beijing, China: Study Protocol,” BMC Public Health 23, no. 1 (2023): 1435. 10.1186/s12889-023-16270-x PMC10373331 37501063

[36] S. J. Landes , S. A. McBain , and G. M. Curran , “An Introduction to Effectiveness‐Implementation Hybrid Designs,” Psychiatry Research 280 (2019): 112513. 10.1016/j.psychres.2019.112513 PMC6779135 31434011

[37] World Health Organization , "Obesity and Overweight," last modified December 8, 2025, https://www.who.int/news‐room/fact‐sheets/detail/obesity‐and‐overweight.

[38] National Health Commission of the People's Republic of China , Guidelines for Myopia Prevention and Control (2024 Edition) (National Health Commission of the People's Republic of China, 2024).

[39] D. I. Flitcroft , M. He , J. B. Jonas , et al., “IMI—Defining and Classifying Myopia: A Proposed Set of Standards for Clinical and Epidemiologic Studies,” Investigative Ophthalmology & Visual Science 60, no. 3 (2019): M20–M30. 10.1167/iovs.18-25957 PMC6735818 30817826

[40] B. J. Smith , A. Grunseit , L. L. Hardy , L. King , L. Wolfenden , and A. Milat , “Parental Influences on Child Physical Activity and Screen Viewing Time: A Population‐Based Study,” BMC Public Health 10 (2010): 593. 10.1186/1471-2458-10-593 PMC3091555 20929587

[41] N. Pearson , S. J. H. Biddle , and T. Gorely , “Family Correlates of Fruit and Vegetable Consumption in Children and Adolescents: A Systematic Review,” Public Health Nutrition 12, no. 2 (2009): 267–283. 10.1017/S1368980008002589 18559129

[42] Y. Qian and P. Lu , “Parents‘ or Guardians’ Knowledge, Attitudes, and Practices in the Prevention and Management of Childhood Myopia,” Ophthalmology and Therapy 13, no. 12 (2024): 3095–3109. 10.1007/s40123-024-01045-3 PMC11564600 39400672

