# Supplementary material for: Effectiveness of a School‐Centered Intervention for Myopia and Obesity Comorbidity in Children: A Cluster‐Randomized Trial
Source: MedComm (2020). 2026 Oct 25;7(11):e71092. doi: 10.1002/mco2.71092 (PMC13695833; doi:10.1002/mco2.71092)
Supplement: Supplementary file 1 [file MCO2-7-e71092-s001.docx]

**Title: Effectiveness of a School-Centered Intervention for Myopia and Obesity Comorbidity in Children: A Cluster-Randomized Trial**

**Running Title: School-Centered Intervention for Myopia and Obesity**

Jing Li^1,2^, Di Shi^1,2^, Yaqi Wang^1,2^, Yihang Zhang^1,2^, Jiajia Dang^1,2^, Yunfei Liu^1,2^, Jieyu Liu^1,2^, Ziyue Chen^1,2^, Xinxin Wang^3^, Jieyun Song^1,2^, Jun Ma^1,2^, Randall S Stafford^4^, Yanhui Dong^1,2,*^, Yi Song^1,2,*^

**Affiliations**:

^1^ Institute of Child and Adolescent Health, School of Public Health, Peking University, Beijing 100191, China

^2^ National Health Commission Key Laboratory of Reproductive Health, Peking University, Beijing 100191, China

^3^ Linyi University, Linyi, Shandong, 276000, China.

^4^Stanford Prevention Research Center, Palo Alto, California, 94304, United States.

**^*^Corresponding authors:**

**Yi Song, MD**

Institute of Child and Adolescent Health, School of Public Health, Peking University

No.38 Xueyuan Road, Haidian District, Beijing 100191, China

E-mail: [songyi@bjmu.edu.cn](mailto:songyi@bjmu.edu.cn)

**Yanhui Dong, MD**

Institute of Child and Adolescent Health, School of Public Health, Peking University

No.38 Xueyuan Road, Haidian District, Beijing 100191, China

E-mail: [dongyanhui@bjmu.edu.cn](mailto:songyi@bjmu.edu.cn)

**Table S1** “22510SS” Behavioral Intervention for Myopia and Obesity Comorbidity in Children and Adolescents

| **Core Concept** | **Specific Components** |
| --- | --- |
| **2** (2-hour outdoor activity) | ≥2 hours of daily outdoor physical activity, including ≥1 hour of moderate-to-vigorous intensity outdoor exercise. |
| **2** (2-hour screen time limit) | ≤2 hours of total daily screen time, with ≤1 hour for recreational electronic device use (each session ≤15 minutes). |
| **5** (5 servings of fruits/vegetables) | Daily intake of 5 adult fist-sized portions (≈300-500 g cooked/chopped leafy vegetables and ≈200-400 g fresh fruits), prioritizing vitamin A/C-rich options (e.g., spinach, bell peppers, carrots, pumpkin). |
| **1** (1 serving of high-quality protein) | Daily 1 palm-sized (80-110 g) portion of lean meat (e.g., beef, fish) or dairy, rich in vitamin A and zinc. |
| **0** (Zero sugary drinks) | Avoid all sugar-sweetened beverages. |
| **S** (Sleep hygiene) | Age-appropriate sleep duration: 10 hours (primary school), 9 hours (junior high). Consistent bedtime/wake-up time; avoid non-sleep activities in bed. |
| **S** (Surveillance & feedback) | Realize the functions of knowledge dissemination, goal setting, behavior monitoring, evaluation feedback, and encouragement and guidance through the application of the applet.  Build a visual and physical index collection and management system, dynamically monitor students’ visual acuity and obesity-related indicators, conduct assessments and provide timely early warnings.  Make a handbook for the prevention and control of myopia and obesity comorbidity, provide education to students, parents, and teachers, train student peer health ambassadors for comorbidity prevention, and enhance students’ sense of ownership. |

Table S2 Topics of the three forms of health education courses

| Type | Topic |
| --- | --- |
| Live-streamed health lectures  (260min in total) | “Embrace Sunshine, Start Healthy” – Webinar 1: The Essentials of 2-Hour Outdoor Activities |
|  | “Love Kids’ Health, Prevent Obesity” – Webinar 2: Key Facts About Childhood Obesity |
|  | “Eat Your Five-a-Day” – Webinar 3: The Power of Daily Fruits & Veggies |
|  | “Grow Healthy, Protein-Powered” – Webinar 4: Daily Quality Protein for Wellness |
|  | “Ditch the Sweet Trap” – Webinar 5: Zero Sugary Drinks Daily |
|  | “Prioritize Sleep, Boost Growth” – Webinar 6: Healthy Sleep Habits for Students |
|  | “Monitor Early, Stay Ahead” – Webinar 7: The Importance of Regular Check-ups |
| Offline health education courses  (40min per school, 18 schools in total) | Protecting Your Vision |
|  | The Double-Edged Sword of Eyeglasses |
|  | Guarding Clear Vision for a Brighter Future |
|  | Cherish Your Eyesight: Be a Vision Guardian! |
|  | Protecting Vision, Building a Brilliant Future |
|  | Stay Healthy, Say No to Obesity |
|  | Small Weight, Big Problem |
|  | Build Exercise Habits, Live a Healthy & Happy Life |
|  | Healthy Weight, Happy Childhood |
|  | Tackling Childhood Obesity for a Healthier School |
| Online health education courses via the WeChat applet  (260min in total) | The Hidden Dangers of Myopia – What You Need to Know |
|  | Understanding the Causes of Myopia – Keeping Kids Glasses-Free! |
|  | Hyperopia in Children – A Parent’s Guide |
|  | Preventing & Managing Childhood Myopia |
|  | Eyeglasses for Myopia – What You Should Know |
|  | Ortho-K Lenses for Myopia – Pros and Cons |
|  | Nutrition and Medication for Myopia Control |
|  | Eye Strain and Accommodative Dysfunction |
|  | Myopia FAQs – Expert Answers |
|  | Understanding Obesity |
|  | Identifying Overweight & Obesity |
|  | The Risks of Childhood Obesity |
|  | Prevention & Health Maintenance |
|  | Intervention & Treatment of obesity |
|  | Debunking Obesity Myths |
|  | How to Read Nutrition Labels |
|  | Healthy Eating Habits |
|  | The Science of Exercise |
|  | The 22510SS Plan – A Practical Guide |

Table S3 Analysis and subgroup analysis of intervention effects on blood lipid and glucose profiles

|  | *β* (95%CI) | *P* | *β*  (95%CI) | *P* |
| --- | --- | --- | --- | --- |
|  | All | | | |
| Serum glucose | -0.05(-0.14,0.05) | 0.334 | / | / |
| High-density lipoprotein cholesterol | 0.01(-0.03,0.04) | 0.726 | / | / |
| Low-density lipoprotein cholesterol | 0.05(-0.00,0.10) | 0.067 | / | / |
| Total Cholesterol | 0.04(-0.04,0.12) | 0.357 | / | / |
| Triglyceride | -0.07(-0.16,0.03) | 0.180 | / | / |
|  | Boys |  | Girls |  |
| Serum glucose | -0.05(-0.15,0.05) | 0.308 | -0.05(-0.15,0.05) | 0.339 |
| High-density lipoprotein cholesterol | 0.02(-0.02,0.07) | 0.260 | -0.01(-0.05,0.04) | 0.764 |
| Low-density lipoprotein cholesterol | 0.04(-0.03,0.10) | 0.269 | 0.06(0.00,0.13) | 0.049 |
| Total Cholesterol | 0.03(-0.07,0.13) | 0.546 | 0.05(-0.05,0.14) | 0.331 |
| Triglyceride | -0.07(-0.17,0.02) | 0.122 | -0.05(-0.15,0.05) | 0.310 |
|  | Grade 1-4 |  | Grade 7 |  |
| Serum glucose | -0.07(-0.19,0.05) | 0.248 | -0.02(-0.18,0.14) | 0.785 |
| High-density lipoprotein cholesterol | 0.01(-0.04,0.06) | 0.607 | -0.00(-0.06,0.06) | 0.913 |
| Low-density lipoprotein cholesterol | 0.04(-0.03,0.11) | 0.221 | 0.06(-0.02,0.14) | 0.135 |
| Total Cholesterol | 0.04(-0.06,0.15) | 0.412 | 0.04(-0.08,0.15) | 0.558 |
| Triglyceride | -0.03(-0.13,0.07) | 0.591 | -0.10(-0.23,0.04) | 0.177 |
|  | Less supportive family environment | | Supportive family environment | |
| Serum glucose | -0.04(-0.13,0.06) | 0.420 | -0.06(-0.17,0.04) | 0.260 |
| High-density lipoprotein cholesterol | 0.01(-0.04,0.05) | 0.772 | 0.01(-0.03,0.05) | 0.588 |
| Low-density lipoprotein cholesterol | 0.08(0.02,0.13) | 0.012 | 0.02(-0.05,0.09) | 0.575 |
| Total Cholesterol | 0.06(-0.02,0.14) | 0.152 | 0.02(-0.09,0.12) | 0.776 |
| Triglyceride | -0.07(-0.17,0.03) | 0.159 | -0.06(-0.15,0.03) | 0.205 |

Note: CI: confidence interval.

Table S4 Incidence of adverse events during the intervention period across different groups

|  | Control group | Intervention group | Χ^2^ | *P* |
| --- | --- | --- | --- | --- |
| Incidence of new-onset underweight | | | 1.116 | 0.291 |
| No | 1566 (96.7) | 1582 (97.4) |  |  |
| Yes | 53 (3.3) | 42 (2.6) |  |  |
| Self-reported bullying related to myopia or obesity during end-term follow up | | | 0.301 | 0.583 |
| No | 1075 (88.8) | 1196 (88.1) |  |  |
| Yes | 135 (11.2) | 162 (11.9) |  |  |

Table S5 Intervention effects on primary effectiveness outcomes by different sex, school stage and family health environment

| Subgroups | Outcome | Control Group | | | Intervention Group | | | Δ1-0 |
| --- | --- | --- | --- | --- | --- | --- | --- | --- |
|  |  | Baseline | End-term follow up | Δ0 | Baseline | End-term follow up | Δ1 |  |
| Boys | SE (D) | -0.88±1.62 | -1.11±1.84 | -0.23 | -1.00±1.61 | -1.32±1.84 | -0.32 | -0.09 (-0.31, 0.14) |
|  | BMI (kg/m^2^) | 19.10±4.15 | 19.25±4.21 | 0.15 | 19.56±4.45 | 19.73±4.48 | 0.17 | 0.02 (-0.52, 0.59) |
|  | Myopia prevalence | 441(51.2) | 496(57.5) | 6.3(1.6,11.0) | 456(53.1) | 536(62.4) | 9.3(4.7,13.9) | 3.0(-3.6,9.6) |
|  | Overweight and obesity prevalence | 427(49.5%) | 394(45.8%) | -3.7(-8.4,1.0) | 448(52.2%) | 415(48.3%) | -3.9(-8.6,0.8) | -0.2(-6.9,6.5) |
|  | Comorbidity prevalence | 217(25.2) | 237(27.5) | 2.3(-1.9,6.5) | 252(29.3) | 275(32.0) | 2.7(-1.6,7.0) | 0.4(-5.6,6.4) |
|  | Overall behavior score of ‘22510SS’ | 4.88±0.96 | 4.73±1.08 | -0.15 | 4.88±1.01 | 4.83±1.04 | -0.05 | 0.10 (-0.09, 0.42) |
| Girls | SE (D) | -0.99±1.62 | -1.21±1.87 | -0.22 | -1.16±1.79 | -1.42±1.91 | -0.26 | -0.04 (-0.26, 0.17) |
|  | BMI (kg/m^2^) | 18.04±3.63 | 18.16±3.77 | 0.12 | 18.14±3.69 | 18.36±3.77 | 0.22 | 0.08 (-0.36,0.51) |
|  | Myopia prevalence | 471(55.9) | 512(60.8) | 4.9(0.2,9.6) | 508(58.9) | 563(65.2) | 6.3(1.7,10.9) | 1.4(-5.1,7.9) |
|  | Overweight and obesity prevalence | 264（31.4%） | 246(29.2) | -2.2(-6.6,2.2) | 270（31.3） | 249(28.9) | -2.4(-6.7,1.9) | -0.2(-6.4,6.0) |
|  | Comorbidity prevalence | 149(17.7) | 156(18.5) | 0.8(-2.9,4.5) | 166(19.2) | 169(19.6) | 0.4(-3.3,4.1) | -0.4(-5.6,4.8) |
|  | Overall behavior score of ‘22510SS’ | 4.79±1.00 | 4.73±1.05 | -0.06 | 4.88±0.99 | 4.84±0.96 | -0.04 | 0.02 (-0.10, 0.34) |
| Grade 1-4 | SE (D) | -0.62±1.24 | -0.86±1.58 | -0.24 | -0.65±1.33 | -0.91±1.51 | -0.26 | -0.02 (-0.17, 0.13) |
|  | BMI (kg/m^2^) | 18.00±3.57 | 18.15±3.74 | 0.15 | 18.03±3.70 | 18.23±3.80 | 0.20 | 0.05 (-0.32, 0.41) |
|  | Myopia prevalence | 673(47.6) | 773(54.7) | 7.1(3.4,10.8) | 624(47.4) | 744(56.5) | 9.1(5.3,12.9) | 2.0(-3.3,7.3) |
|  | Overweight and obesity prevalence | 570(40.3%) | 533(37.7%) | -2.6(-6.2,1.0) | 536(40.7) | 506(38.4) | -2.3(-6.0,1.4) | 0.3(-4.9,5.5) |
|  | Comorbidity prevalence | 270(19.1) | 307(21.7) | 2.6(-0.4,5.6) | 267(20.3) | 305(23.2) | 2.9(-0.2,6.0) | 0.3(-4.0,4.6) |
|  | Overall behavior score of ‘22510SS’ | 4.83±0.97 | 4.76±1.04 | -0.07 | 4.91±0.96 | 4.87±0.98 | -0.04 | 0.03 (-0.07, 0.32) |
| Grade 7 | SE (D) | -2.46±2.28 | -2.61±2.35 | -0.15 | -2.48±2.01 | -2.86±2.16 | -0.38 | -0.23 (-0.38, -0.08) |
|  | BMI (kg/m^2^) | 21.36±4.42 | 21.44±4.30 | 0.12 | 21.51±4.40 | 21.67±4.35 | 0.16 | 0.04 (-0.79, 1.00) |
|  | Myopia prevalence | 239(82.4) | 235(81.0) | -1.4(-7.7,4.9) | 340(83.7) | 355(87.4) | 3.7(-1.1,8.5) | 5.1(-2.8,13.0) |
|  | Overweight and obesity prevalence | 121(41.7%) | 107(36.9%) | -4.8(-12.7,3.1) | 182(44.9) | 158(38.9) | -6.0(-12.7,0.7) | -1.2(-11.6,9.2) |
|  | Comorbidity prevalence | 96(33.1) | 86(29.7) | -3.4(-10.9,4.1) | 151(37.2) | 139(34.2) | -3.0(-9.6,3.6) | 0.4(-9.6,10.4) |
|  | Overall behavior score of ‘22510SS’ | 4.86±1.04 | 4.62±1.15 | -0.24 | 4.76±1.10 | 4.73±1.05 | -0.03 | 0.21 (-0.17, 0.49) |
| Less supportive family environment | SE (D) | -0.95±1.64 | -1.15±1.84 | -0.20 | -1.09±1.74 | -1.37±1.86 | -0.28 | -0.08 (-0.30, 0.13) |
|  | BMI (kg/m^2^) | 18.60±4.06 | 18.72±4.15 | 0.12 | 19.06±4.23 | 19.27±4.27 | 0.21 | 0.09 (-0.45, 0.53) |
|  | Myopia prevalence | 412(55.2) | 446(59.8) | 4.6(-0.4,9.6) | 474(54.2) | 542(61.9) | 7.7(3.1,12.3) | 3.1(-3.7,9.9) |
|  | Overweight and obesity prevalence | 296(39.7%) | 275(36.9) | -2.8(-7.7,2.1) | 375(42.8) | 353(40.3) | -2.5(-7.1,2.1) | 0.3(-6.4,7.0) |
|  | Comorbidity prevalence | 165(22.1) | 174(23.3) | 1.2(-3.0,5.4) | 206(23.5) | 224(25.6) | 2.1(-1.9,6.1) | 0.9(-4.9,6.7) |
|  | Overall behavior score of ‘22510SS’ | 4.82±0.95 | 4.68±1.05 | -0.14 | 4.85±0.99 | 4.80±1.00 | -0.05 | 0.09 (-0.09, 0.39) |
| Supportive family environment | SE (D) | -0.96±1.66 | -1.18±1.95 | -0.22 | -1.06±1.63 | -1.32±1.79 | -0.26 | -0.04 (-0.29, 0.21) |
|  | BMI (kg/m^2^) | 18.53±3.84 | 18.65±3.95 | 0.12 | 18.68±4.07 | 18.87±4.10 | 0.19 | 0.07 (-0.43, 0.58) |
|  | Myopia prevalence | 380(54.0) | 414(58.8) | 4.8(-0.4,10.0) | 418(57.5) | 471(64.8) | 7.3(2.3,12.3) | 2.5(-4.7,9.7) |
|  | Overweight and obesity prevalence | 287(40.8%) | 264(37.5) | -3.3(-8.4,1.8) | 298(41.0) | 272(37.4) | -3.6(-8.6,1.4) | -0.3(-7.4,6.8) |
|  | Comorbidity prevalence | 152(21.6) | 161(22.9) | 1.3(-3.0,5.6) | 180(24.8) | 191(26.3) | 1.5(-3.0,6.0) | 0.2(-6.0,6.4) |
|  | Overall behavior score of ‘22510SS’ | 4.90±0.97 | 4.79±1.00 | -0.11 | 4.91±1.02 | 4.89±0.97 | -0.02 | 0.09 (-0.11, 0.40) |

Note:SE: spherical equivalent, BMI: Body mass index.

Table S6 Subgroup analysis of intervention effects on secondary outcomes

|  | OR (95%CI) | *P* | OR (95%CI) | *P* | *P_interaction_* |
| --- | --- | --- | --- | --- | --- |
|  | Boys |  | Girls |  |  |
| Incidence rate of myopia | 1.00(0.61,1.67) | 0.986 | 1.05(0.53,2.07) | 0.884 | 0.924 |
| Incidence rate of OWOB | 0.56(0.21,1.46) | 0.236 | 0.73(0.38,1.42) | 0.359 | 0.564 |
| Incidence rate of comorbidity | 1.00(0.60,1.66) | 0.998 | 0.96(0.48,1.93) | 0.905 | 0.731 |
| Progression rate of myopia | 0.80(0.35,1.82) | 0.594 | 0.72(0.46,1.13) | 0.153 | 0.397 |
| Progression rate of OWOB | 1.11(0.43,2.85) | 0.825 | 0.74(0.13,4.04) | 0.724 | 0.764 |
| Overall behavior improvement rate | 1.33(0.98,1.80) | 0.066 | 1.27(0.88,1.84) | 0.196 | 0.668 |
|  | Grade 1-4 |  | Grade 7 |  |  |
| Incidence rate of myopia | 0.99(0.52,1.86) | 0.965 | 1.23(0.47,3.22) | 0.667 | 0.933 |
| Incidence rate of OWOB | 0.73(0.36,1.49) | 0.386 | 0.15(0.01,2.27) | 0.172 | 0.073 |
| Incidence rate of comorbidity | 1.10(0.62,1.95) | 0.738 | 0.69(0.21,2.22) | 0.532 | **0.041** |
| Progression rate of myopia | 0.46(0.25,0.86) | **0.014** | 1.33(0.79,2.26) | 0.287 | **0.023** |
| Progression rate of OWOB | 0.83(0.32,2.14) | 0.701 | 6.46(0.27,153.78) | 0.249 | 0.828 |
| Overall behavior improvement rate | 1.19(0.85,1.65) | 0.307 | 1.84(1.02,3.33) | **0.044** | 0.863 |
|  | Less supportive family environment | | Supportive family environment | |  |
| Incidence rate of myopia | 0.99(0.55,1.79) | 0.978 | 1.07(0.58,1.96) | 0.831 | 0.866 |
| Incidence rate of OWOB | 0.43(0.21,0.90) | **0.024** | 1.05(0.44,2.52) | 0.913 | **0.038** |
| Incidence rate of comorbidity | 0.97(0.54,1.72) | 0.909 | 1.03(0.57,1.86) | 0.926 | 0.522 |
| Progression rate of myopia | 0.51(0.32,0.83) | **0.006** | 1.19(0.67,2.11) | 0.546 | **0.013** |
| Progression rate of OWOB | 0.96(0.30,3.09) | 0.947 | 0.84(0.31,2.26) | 0.733 | 0.735 |
| Overall behavior improvement rate | 1.41(1.02,1.95) | **0.038** | 1.23(0.88,1.72) | 0.229 | 0.622 |

Note: OWOB: overweight and obesity; OR: odds ratio; CI: confidence interval.

Table S7 Analysis of intervention effects on each “22510SS” behavior

| Subgroups | Outcome | Control Group | | | Intervention Group | | | Δ1-0 |
| --- | --- | --- | --- | --- | --- | --- | --- | --- |
|  |  | Baseline | End-term follow up | Δ0 | Baseline | End-term follow up | Δ1 |  |
| All | Achievement rate of ‘2-outdoor activity’ | 428(34.8) | 338(28.7) | -6.1 (-9.9, -2.5) | 487(36.1) | 432(32.0) | -4.1(-7.6, -0.5) | 2.0 (-3.0, 7.2) |
|  | Achievement rate of ‘2-screen time’ | 935(68.5) | 950(72.8) | 4.4 (0.9, 7.8) | 1040(70.7) | 1096(75.3) | 4.6 (1.3, 7.7) | 0.2 (-4.5, 4.9) |
|  | Achievement rate of ‘5/1-healthy eating’ | 435(27.7) | 481(35.6) | 7.9 (4.5, 11.2) | 439(26.9) | 488(32.4) | 5.5 (2.3, 8.7) | -2.4 (-7.0, 2.3) |
|  | Achievement rate of ‘0-SSBs’ | 484(30.8) | 510(37.6) | 6.8 (3.3, 10.2) | 497(30.5) | 605(39.9) | 9.4 (6.1, 12.8) | 2.6 (-2.1, 7.5) |
|  | Achievement rate of ‘S-sleep duration’ | 356(23.5) | 340(25.8) | 2.3 (-1.0, 5.4) | 305(19.6) | 344(23.2) | 3.6 (0.7, 6.5) | 1.3 (-2.9, 5.7) |
|  | Achievement rate of ‘S-supervised regularly’ | — | 296 (22.0) | — | — | 399 (26.5) | — | — |
| Boys | Achievement rate of ‘2-outdoor activity’ | 240(38.3) | 172(30.3) | -8.0(-13.3,-2.7) | 256(37.6) | 244(36.5) | -1.1(-6.2,4.0) | 6.9(-0.5,14.3) |
|  | Achievement rate of ‘2-screen time’ | 475(69.0) | 465(73.6) | 4.6(-0.3,9.5) | 531(71.8) | 558(77.1) | 5.3(0.8,9.8) | 0.7(-5.9,7.3) |
|  | Achievement rate of ‘5 /1 -healthy eating’ | 218(27.6) | 249(37.9) | 10.3(5.5,15.1) | 250(30.8) | 255(34.4) | 3.6(-1.1,8.3) | -6.7(-13.4,0.0) |
|  | Achievement rate of ‘0-SSBs’ | 233(29.6) | 243(36.7) | 7.1(2.2,12.0) | 236(29.0) | 293(39.1) | 10.1(5.4,14.8) | 3.0(-3.7,9.7) |
|  | Achievement rate of ‘S-sleep duration’ | 187(24.5) | 170(26.6) | 2.1(-2.5,6.7) | 168(21.7) | 184(25.0) | 3.3(-1.0,7.6) | 1.2(-5.1,7.5) |
|  | Achievement rate of ‘S-supervised regularly’ | 131(16.5) | 151(22.9) | 6.4(2.3,10.5) | 140(17.3) | 196(26.1) | 8.8(4.7,12.9) | 2.4(-3.4,8.2) |
| Girls | Achievement rate of ‘2-outdoor activity’ | 188(31.2) | 166(27.2) | -4.0(-9.1,1.1) | 231(34.6) | 188(27.7) | -6.9(-11.8,-2.0) | -2.9(-10.0,4.2) |
|  | Achievement rate of ‘2-screen time’ | 460(67.9) | 485(72.2) | 4.3(-0.6,9.2) | 509(69.7) | 538(73.5) | 3.8(-0.8,8.4) | -0.5(-7.2,6.2) |
|  | Achievement rate of ‘5 /1 -healthy eating’ | 217(27.9) | 232(33.4) | 5.5(0.8,10.2) | 189(23.0) | 233(30.5) | 7.5(3.2,11.8) | 2.0(-4.4,8.4) |
|  | Achievement rate of ‘0-SSBs’ | 251(32.0) | 267(38.5) | 6.5(1.6,11.4) | 261(31.9) | 312(40.7) | 8.8(4.1,13.5) | 2.3(-4.5,9.1) |
|  | Achievement rate of ‘S-sleep duration’ | 169(22.5) | 170(25.0) | 2.5(-1.9,6.9) | 137(17.5) | 160(21.4) | 3.9(-0.1,7.9) | 1.4(-4.5,7.3) |
|  | Achievement rate of ‘S-supervised regularly’ | 114(14.5) | 145(21.2) | 6.7(2.8,10.6) | 144(17.5) | 203(26.9) | 9.4(5.3,13.5) | 2.7(-3.0,8.4) |
| Grade 1-4 | Achievement rate of ‘2-outdoor activity’ | 322(32.7) | 258(27.4) | -5.3(-9.4,-1.2) | 313(31.6) | 285(28.6) | -3.0(-7.0,1.0) | 2.3(-3.4,8.0) |
|  | Achievement rate of ‘2-screen time’ | 802(72.5) | 791(76.1) | 3.6(-0.1,7.3) | 821(75.7) | 863(79.5) | 3.8(0.3,7.3) | 0.2(-4.9,5.3) |
|  | Achievement rate of ‘5 /1 -healthy eating’ | 345(26.7) | 371(34.3) | 7.6(3.9,11.3) | 298(24.1) | 346(30.7) | 6.6(3.0,10.2) | -1.0(-6.2,4.2) |
|  | Achievement rate of ‘0-SSBs’ | 419(32.3) | 442(40.7) | 8.4(4.5,12.3) | 392(31.7) | 482(42.5) | 10.8(6.9,14.7) | 2.4(-3.1,7.9) |
|  | Achievement rate of ‘S-sleep duration’ | 271(21.6) | 277(26.0) | 4.4(0.9,7.9) | 214(18.0) | 245(21.8) | 3.8(0.5,7.1) | -0.6(-5.4,4.2) |
|  | Achievement rate of ‘S-supervised regularly’ | 201(15.4) | 234(21.8) | 6.4(3.2,9.6) | 195(15.8) | 282(25.0) | 9.2(6.0,12.4) | 2.8(-1.7,7.3) |
| Grade 7 | Achievement rate of ‘2-outdoor activity’ | 106(43.6) | 80(33.9) | -9.7(-18.3,-1.1) | 174(48.6) | 147(41.6) | -7.0(-14.3,0.3) | 2.7(-8.6,14.0) |
|  | Achievement rate of ‘2-screen time’ | 133(51.4) | 159(60.2) | 8.8(0.4,17.2) | 219(56.7) | 233(63.0) | 6.3(-0.6,13.2) | -2.5(-13.4,8.4) |
|  | Achievement rate of ‘5 /1 -healthy eating’ | 90(32.8) | 110(40.9) | 8.1(0.1,16.1) | 141(35.8) | 142(37.5) | 1.7(-5.1,8.5) | -6.4(-16.9,4.1) |
|  | Achievement rate of ‘0-SSBs’ | 65(23.8) | 68(25.3) | 1.5(-5.7,8.7) | 105(26.6) | 123(32.3) | 5.7(-0.7,12.1) | 4.2(-5.4,13.8) |
|  | Achievement rate of ‘S-sleep duration’ | 85(32.8) | 63(24.8) | -8.0(-15.8,-0.2) | 91(24.7) | 99(27.7) | 3.0(-3.4,9.4) | **11.0(1.0,21.0)** |
|  | Achievement rate of ‘S-supervised regularly’ | 44(16.1) | 62(23.0) | 6.9(0.3,13.5) | 89(22.6) | 117(31.0) | 8.4(2.2,14.6) | 1.5(-7.6,10.6) |
| Less supportive family environment | Achievement rate of ‘2-outdoor activity’ | 179(31.2) | 140(27.2) | -4.0(-9.4,1.4) | 235(33.5) | 206(29.3) | -4.2(-9.0,0.6) | -0.2(-7.4,7.0) |
|  | Achievement rate of ‘2-screen time’ | 421(68.0) | 419(73.1) | 5.1(-0.1,10.3) | 516(67.8) | 559(74.2) | 6.4(1.9,10.9) | 1.3(-5.6,8.2) |
|  | Achievement rate of ‘5 /1 -healthy eating’ | 181(25.1) | 195(32.9) | 7.8(2.9,12.7) | 217(25.5) | 226(28.9) | 3.4(-0.9,7.7) | -4.4(-10.9,2.1) |
|  | Achievement rate of ‘0-SSBs’ | 196(27.1) | 220(37.2) | 10.1(5.0,15.2) | 243(28.6) | 310(39.5) | 10.9(6.3,15.5) | 0.8(-6.0,7.6) |
|  | Achievement rate of ‘S-sleep duration’ | 163(23.5) | 135(23.3) | -0.2(-4.9,4.5) | 154(18.9) | 166(21.6) | 2.7(-1.3,6.7) | 2.9(-3.2,9.0) |
|  | Achievement rate of ‘S-supervised regularly’ | 103(14.2) | 125(21.3) | 7.1(2.9,11.3) | 144(16.9) | 214(27.5) | 10.6(6.6,14.6) | 3.5(-2.3,9.3) |
| Supportive family environment | Achievement rate of ‘2-outdoor activity’ | 208(38.1) | 148(29.4) | -8.7(-14.4,-3.0) | 236(40.3) | 208(37.2) | -3.1(-8.7,2.5) | 5.6(-2.4,13.6) |
|  | Achievement rate of ‘2-screen time’ | 420(69.2) | 405(73.2) | 4.0(-1.2,9.2) | 477(74.8) | 469(77.1) | 2.3(-2.4,7.0) | -1.7(-8.7,5.3) |
|  | Achievement rate of ‘5 /1 -healthy eating’ | 202(29.5) | 206(36.1) | 6.6(1.4,11.8) | 205(29.0) | 237(37.6) | 8.6(3.6,13.6) | 2.0(-5.2,9.2) |
|  | Achievement rate of ‘0-SSBs’ | 234(34.3) | 221(38.5) | 4.2(-1.1,9.5) | 233(32.9) | 257(40.7) | 7.8(2.7,12.9) | 3.6(-3.8,11.0) |
|  | Achievement rate of ‘S-sleep duration’ | 163(24.7) | 142(25.5) | 0.8(-4.1,5.7) | 135(19.9) | 151(24.3) | 4.4(-0.1,8.9) | 3.6(-3.0,10.2) |
|  | Achievement rate of ‘S-supervised regularly’ | 117(17.0) | 129(22.8) | 5.8(1.4,10.2) | 125(17.7) | 163(26.0) | 8.3(3.9,12.7) | 2.5(-3.8,8.8) |

Figure S1 Participant flow and reasons for loss to follow-up

Figure S2 Effectiveness in Overall and Individual “22510SS” Behavioral Indicators

**Appendix A. Delphi method-based implementation research outcome indicator construction**

**1.1 Initial construction of the indicator set**

In this study, we used a step-by-step deductive approach, combining the literature and the actual research needs, and initially constructed a set of outcome indicators for the implementation of integrated intervention for myopia and obesity co-morbidity in children and adolescents.

Firstly, the RE-AIM framework was used as the basis for the five dimensions of the RE-AIM framework (reach, effectiveness, adoption, implementation, and maintenance) as first-level indicators, and according to the guidance for evaluating each of the dimensions provided on the RE-AIM website, the meaning of each dimension was clearly defined in the context of the implementation study of comprehensive intervention for childhood and adolescent myopia and obesity co-morbidities, as shown in **Table A1.** Secondly, based on the definition of the first-level indicators, the second-level indicators were further refined, and according to the needs of the study, the third-level indicators were also set up under some of the second-level indicators. Finally, specific calculation methods, data collection time and data sources were added for each indicator.

**Table A1. RE-AIM assessment framework for the outcome indicators of the comprehensive intervention study for multimorbidity of myopia and obesity among childhood and adolescents**

| Assessment dimensions | Meaning | Level of assessment |
| --- | --- | --- |
| Reach | Number, proportion and representativeness of children and adolescents who met the inclusion and exclusion criteria to participate in the study. | individually |
| Effectiveness | Effectiveness of the interventions among participants. | individually |
| Adoption | Institutions (schools, health clinics or CDCs) involved in the project and number, proportion and representation of staff (school physician, health clinic or CDC staff). | individually organizations |
| Implementation | The extent to which work has progressed in accordance with the project plan, the adjustments made to the process of implementing the content of the intervention, and the cost of implementing the intervention in the project. | organizations |
| Maintenance | The extent to which the intervention was sustained after the project ended, and the impact of implementation on individual health. | individually |

**1.2 Selection of experts**

A total of 28 experts were recruited, representing diverse fields such as pediatrics, ophthalmology, endocrinology, as well as school doctors, teachers, and officials from the CDC and healthcare institutions. Their multidisciplinary input contributed to the comprehensive development of the evaluation indicators. Several experts were also familiar with the study’s intervention program and the practical needs in the field, helping to ensure that the indicator development was closely aligned with the study’s objectives and maintained both scientific and practical value. The basic characteristics of the participating experts are presented in **Table A2.**

**Table A2. Basic information of experts**

| **Variables** | **N (%)** |
| --- | --- |
| Gender |  |
| Male | 15(53.6) |
| Female | 13(46.4) |
| Working experience |  |
| < 10 years | 8(28.6) |
| 10-20 years | 9(32.1) |
| > 20 years | 11(39.3) |
| Major |  |
| Child and adolescent health | 8(28.6) |
| Clinical medicine | 2(7.1) |
| Health management | 4(14.3) |
| School nurse | 6(21.4) |
| Teacher | 8(28.6) |
| Education |  |
| PhD | 6(21.4) |
| Master | 2(7.1) |
| Bachelor | 18(64.4) |
| Junior college | 2(7.1) |
| Title |  |
| Senior | 2(7.1) |
| Deputy senior | 5(17.9) |
| Intermediate | 13(46.4) |
| Junior | 4(14.3) |
| None | 4(14.3) |

**1.3 Expert Consultation**

A total of two rounds of expert consultation were conducted. The first-round questionnaire included the following four sections: (1) Introduction: An overview of the study’s background, objectives, and methodology; (2) Expert Information Survey: Collection of data on experts’ educational background, professional qualifications, fields of expertise, and years of experience; (3) Questionnaire Instructions: Explanation of the study purpose and the scoring procedure; (4) Consultation Form: Presentation of the initial set of indicators, including definitions, calculation methods, and data sources. Experts rated each indicator using a five-point Likert scale (1 = not important to 5 = very important), and were invited to provide suggestions and indicate their familiarity and judgment basis.

The second-round questionnaire, revised based on feedback from the first round, consisted of five sections: (1) Introduction: A brief summary of the study’s background, objectives, and methods; (2) Expert Information Survey: Same as in the first round; (3) Results of the First Round: Summary statistics for each indicator, including mean score, standard deviation, ranking, and any revisions; (4) Questionnaire Instructions: Guidelines for completing the second-round evaluation; (5) Consultation Form: Experts re-evaluated the revised indicators, provided updated ratings and suggestions, and again reported their familiarity and judgment basis.

1.4 **Statistical analysis**

Based on experts’ importance ratings for each indicator, four evaluation metrics were calculated to assess the quality and reliability of the consultation process. The expert positivity index was assessed by the questionnaire response rate, calculated as the number of experts who completed the survey divided by the total number invited.

The expert authority coefficient ($C_{r}$) was calculated to evaluate the credibility of expert judgments and was defined as the average of two components: the judgment basis coefficient ($C_{a}$) and the familiarity coefficient ($C_{s}$), i.e., $C_{r}$ = ($C_{a}$+ $C_{s}$)/2. The judgment basis coefficient ($C_{a}$) reflects the types of evidence relied upon by the expert when making evaluations, including theoretical analysis, practical experience, understanding of peer practices, and intuition. Each judgment basis was assigned a weight according to its level of influence, typically ranging from 0.1 to 0.5., as shown in Table A3. The familiarity coefficient ($C_{s}$) was self-reported by each expert and reflected their familiarity with the evaluated indicators. It was rated on a five-point scale: very familiar (1.0), fairly familiar (0.8), generally familiar (0.6), somewhat unfamiliar (0.4), and unfamiliar (0.2).

**Table A3. Assignment Values for Expert Judgment Criteria**

| Judgment Basis | High Influence | Medium Influence | Low Influence |
| --- | --- | --- | --- |
| Theoretical analysis | 0.30 | 0.20 | 0.10 |
| Practical experience | 0.50 | 0.40 | 0.30 |
| Understanding of peer practices | 0.10 | 0.10 | 0.05 |
| Intuition | 0.10 | 0.10 | 0.05 |

The coordination of expert opinions was assessed using the coefficient of variation (*V****_j_***) and Kendall’s coefficient of concordance (W). The coefficient of variation reflects the degree of consensus among experts regarding a specific indicator, where a lower value indicates better agreement. The Kendall’s W measures the overall consistency of expert opinions across all indicators and reflects the reliability of the consultation results. The significance of W was tested using a chi-square test based on the degrees of freedom, with a significance level of α = 0.05 and critical value χ²_α_. If the test statistic χ²_α_ ＞χ²_α_ (where R is the number of experts rating the indicators), the result is considered statistically significant (P < 0.05), indicating good coordination among expert opinions and valid results.The coefficient of variation was calculated using Equation:

$$V_{j}= \frac{\sigma_{j}}{M_{j}}$$

where σ_j_ is the standard deviation of expert ratings for indicator, and *M_j_* is the mean score of indicators.

The concentration of expert opinions was evaluated using the mean score ($M_{j}$), full score frequency ($K_{j}$), and the sum of assigned scores (***S_j_*)**. These were calculated using following Equations. Higher values of these indicators reflect greater consensus and higher perceived importance of the indicator.

$$M_{j}=\frac{1}{m}\sum_{i=1}^{m} C_{ij}$$

$K_{j}$= (Number of experts giving full score to indicator) / (Total number of experts rating indicator) × 100%

Following confirmation of rating stability, indicators were excluded if they ranked low in importance, showed high variability in expert ratings (coefficient of variation > 0.2), or had low mean scores (mean < 3.5). All statistical analyses were conducted using SPSS 27.0 and R 4.4.0, with a two-tailed significance level set at α = 0.05.

1.5 **Results of expert consultation**

**Results of the First Round of Expert Consultation and Revisions**

In the first round of expert consultation, importance ratings were collected for 16 secondary indicators and 43 tertiary indicators under five preliminarily established primary domains. These domains and indicators included:

(1) **Reach:** number of children and adolescents participating in the program; proportion of participants relative to the target population; and representativeness of participants (including those who completed the full intervention).
(2) **Effectiveness:** primary disease outcomes (prevalence of comorbidities); secondary outcomes such as new incidence of myopia, equivalent spherical refractive error, body mass index (BMI), waist circumference, and prevalence of overweight and obesity; levels of health knowledge (e.g., 2 hours of outdoor activity per day, ≤2 hours of screen time, 5 servings of fruits and vegetables, 1 serving of high-quality protein, no sugary drinks, adequate sleep, and regular health monitoring); levels of health behavior (including physical activity, screen and eye-use behavior, dietary behavior, sleep behavior, and monitoring behavior); and adverse outcomes such as wasting/stunting rates.
(3) **Adoption:** institutional-level indicators (e.g., proportion and representativeness of schools involved); and individual-level indicators (e.g., number and representativeness of school doctors and staff from health centers or disease control institutions involved in the project).
(4) **Implementation:** fidelity, number and rationale for content adjustments, and associated costs (total cost, per capita cost, incremental cost, and cost-effectiveness).
(5) **Maintenance:** individual health outcomes (e.g., prevalence of comorbidities, new cases of myopia, overweight and obesity); and institutional sustainability (e.g., proportion of schools continuing implementation, retention of project components, and interest from new institutions).

The analysis showed that mean indicator scores ranged from 4.18 to 5.00, with coefficients of variation ranging from 0.00 to 0.25, and full-score frequencies ranging from 50.0% to 100.0%. Expert feedback included recommendations to clarify indicator definitions and calculation methods, revise or remove indicators with low feasibility, and adjust content as necessary. Based on these suggestions, the indicator system was revised: two primary indicator definitions were modified; three secondary indicators were revised and two were removed; and five tertiary indicators were revised while two were deleted.

**Results of the Second Round of Expert Consultation and Revisions**

The second round of expert consultation evaluated 13 secondary indicators and 30 tertiary indicators under the revised five primary domains. The updated indicator set included:

(1) **Reach:** number of participants and their representativeness (including those who completed the program).
(2) **Effectiveness:** primary disease outcomes (incidence of comorbidities); secondary outcomes (including new incidence of myopia, equivalent spherical refractive error, BMI, waist-to-height ratio, and prevalence of overweight and obesity); health knowledge indicators (same seven items as in the first round); and health behavior indicators (composite health behavior scores).
(3) **Adoption:** institutional-level representativeness of schools; and individual-level representativeness of school doctors and teachers involved.
(4) **Implementation:** fidelity, number and reasons for content adjustments, and cost-related indicators (total cost, per capita cost, incremental cost, and cost-effectiveness).
(5) **Maintenance:** individual health outcomes (prevalence of comorbidities, new incidence of myopia, overweight and obesity); and institutional sustainability, assessed using a project continuity evaluation tool.

The results showed that mean scores ranged from 4.11 to 5.00, coefficients of variation from 0.00 to 0.13, and full-score frequencies from 14.3% to 100.0%. Experts recommended merging similar indicators with low importance ratings, clarifying indicator definitions, and making other refinements. As a result, four secondary indicators and four tertiary indicators were modified.

The final refined indicator system comprises 5 primary indicators, 13 secondary indicators, and 20 tertiary indicators. Details of the final indicators, including their mean scores, standard deviations, coefficients of variation, and full-score frequencies, are presented in Table A4.

**Table A4. Indicators of the implementation study of an integrated intervention for myopia and obesity co-morbidity in children and adolescents based on the RE-AIM framework.**

| **Primary indicator** | **Secondary indicator** | **Tertiary indicator** | **Mean** | **Standard deviation** | **Coefficient of variation** | **Full score frequency (%)** |
| --- | --- | --- | --- | --- | --- | --- |
| Reach | Number of children and adolescents enrolled |  | 4.86 | 0.36 | 0.07 | 85.7 |
|  | Representativeness of participants | Baseline representativeness | 4.96 | 0.19 | 0.04 | 96.4 |
|  |  | Full-course representativeness | 4.93 | 0.26 | 0.05 | 92.9 |
| Effectiveness | Primary disease outcomes | Incidence of multimorbid conditions | 5.00 | 0.00 | 0.00 | 100.0 |
|  | Secondary disease outcomes | Incidence rate of myopia | 5.00 | 0.00 | 0.00 | 100.0 |
|  |  | Spherical equivalent | 4.89 | 0.31 | 0.06 | 89.3 |
|  |  | Body mass index | 4.96 | 0.19 | 0.04 | 96.4 |
|  |  | Prevalence of overweight and obesity | 4.93 | 0.26 | 0.05 | 92.9 |
|  |  | Waist-to-height ratio | 5.00 | 0.00 | 0.00 | 100.0 |
|  | Health literacy | Comprehensive health knowledge score | 4.59 | 0.43 | 0.10 | 61.2 |
|  | Health behaviors | Comprehensive health behavior score | 4.93 | 0.26 | 0.05 | 92.9 |
| Adoption | Organizational level | Representativeness of participating schools | 4.86 | 0.36 | 0.07 | 85.7 |
|  | Individual level | Representativeness of school nurses/teachers | 4.75 | 0.44 | 0.09 | 75.0 |
| Implementation | Fidelity |  | 4.96 | 0.19 | 0.04 | 96.4 |
|  | Content modifications |  | 4.31 | 0.47 | 0.11 | 30.35 |
|  | Cost |  | 4.50 | 0.48 | 0.11 | 52.70 |
| Maintenance | Individual health outcomes | Prevalence of comorbid conditions | 5.00 | 0.00 | 0.00 | 100.0 |
|  |  | Prevalence of myopia | 4.96 | 0.19 | 0.04 | 96.4 |
|  |  | Prevalence of overweight and obesity | 5.00 | 0.00 | 0.00 | 100.0 |
|  | Organizational sustainment |  | 4.93 | 0.26 | 0.05 | 92.9 |

The calculation of the mean, standard deviation, coefficient of variation, and full score frequency was based on the results from the second round of expert consultation.

Appendix B. Measurements of socioeconomic status.

| **Field** | **Item** | **Processing** | **Coding** | **Coding notes** |
| --- | --- | --- | --- | --- |
| **Demographics** | Maternal Education | Categorical | Bachelor's degree or above | Set as 0 |
|  |  |  | High school or below | Set as 1 |
|  |  |  | NA | Set as NA |
|  | Paternal occupation | Categorical | Housework | Set as 1, unemployed |
|  |  |  | Unemployed/Laid-off | Set as 1, unemployed |
|  |  |  | Retired personnel | Set as 1, unemployed |
|  |  |  | Head of government, party, or business units | Set as 0, employed |
|  |  |  | Professional and technical staff (e.g., teachers, doctors) | Set as 0, employed |
|  |  |  | Commercial or service industry personnel | Set as 0, employed |
|  |  |  | Clerical and related staff (e.g., police officers, postal and telecommunication workers) | Set as 0, employed |
|  |  |  | Agricultural, forestry, animal husbandry, fishery, and water conservancy workers | Set as 0, employed |
|  |  |  | Production, transport equipment operators, and related personnel | Set as 0, employed |
|  |  |  | Military personnel | Set as 0, employed |
|  |  |  | Other laborers | Set as 0, employed |
|  |  |  | NA | Set as NA |

Appendix C. RE-AIM framework outcomes

C.1.1 Reach Dimension Methods

The RE-AIM Reach dimension was assessed through participant enrollment counts and representativeness analyses. Participation was defined as the number of eligible children and adolescents who provided informed consent. Representativeness was evaluated by comparing participants' baseline characteristics including sex, age, vision status and nutritional status with reference data from Beijing and national pediatric populations, as well as comparing baseline characteristics between study completers and the original cohort.

Demographic data were collected through standardized questionnaires, while vision and nutritional status were obtained through physical examinations. Reference population data came from official education statistics published by the Beijing Municipal Education Commission and the Ministry of Education, supplemented by national student health surveillance data. Statistical analyses employed χ² tests to examine differences between: 1) study participants and reference populations in terms of demographic composition, myopia prevalence, obesity rates and comorbidity rates; and 2) baseline participants versus study completers versus dropouts.

C.1.2 Reach Dimension Results

The research enrolled 4,991 children and adolescents at baseline, representing 2.6% of eligible students in grades 1–4 and 7 across three districts (Dongcheng, Tongzhou, Changping) in Beijing. Of these, 3,426 (68.6%) completed the 12-month follow-up, with 1,565 (31.4%) lost to attrition.

Compared with the gender and school stage ratios in Beijing reported by the Beijing Municipal Education Commission, there was no significant difference in gender composition, and there was a significant difference in the composition ratio of school stage. Compared with the prevalence of myopia, overweight and obesity and comorbidities in Beijing, there were significant differences. See Figure C1.

Figure C1 Representativeness of participants in this research project

The attrition analysis revealed significant differences between participants lost to follow-up and those completing follow-up in age, grade distribution, nutritional status, and BMI (all P<0.001). Participants lost to follow-up were younger and had lower baseline BMI, and completion was lowest in grade 1 and highest in grade 7. Sex, single-child status, and socioeconomic status showed no significant differences (P>0.05). Baseline myopia and nutritional status also differed significantly between groups, whereas comorbidity prevalence was similar (P=0.865). These patterns suggest that attrition may have affected the representativeness of the analytic sample. See Table C1.

Table C1 Comparison of baseline characteristics of missing samples across groups and between missing and follow-up samples

| Characteristic | | Missing sample | | | | Completed follow-up sample  (n= 3426) | *P* |
| --- | --- | --- | --- | --- | --- | --- | --- |
|  |  | Control group | Intervention group | *P* | Total missing |  |  |
|  |  | (n=696) | (n=869) |  | (n=1565) |  |  |
| Age (Y) | | 8.91[7.91,10.09] | 8.22[7.10,9.68] | **<0.001** | 8.55[7.37,9.85] | 8.89[7.85,10.24] | **<0.001** |
| Sex | |  |  | 0.915 |  |  | 0.120 |
|  | Boys | 368(44.7) | 456(55.3) |  | 824(32.5) | 1721(67.5) |  |
|  | Girls | 328(44.3) | 413(55.7) |  | 741(30.3) | 1705(69.7) |  |
| Grade | |  |  | **<0.001** |  |  | **<0.001** |
|  | Grade 1 | 124(32.0) | 264(68.0) |  | 388(38.8) | 613(61.2) |  |
|  | Grade 2 | 132(41.3) | 188(58.8) |  | 320(30.7) | 724(69.3) |  |
|  | Grade 3 | 159(50.8) | 154(49.2) |  | 313(29.6) | 742(70.4) |  |
|  | Grade 4 | 165(54.3) | 139(45.7) |  | 304(31.8) | 651(68.2) |  |
|  | Grade 7 | 116(48.3) | 124(51.7) |  | 240(25.7) | 696(74.3) |  |
| Single child status | | |  | 0.738 |  |  | 0.071 |
|  | Yes | 273(50.1) | 272(49.9) |  | 545(24.2) | 1714(75.8) |  |
|  | No | 198(51.5) | 187(48.5) |  | 385(21.7) | 1392(78.3) |  |
| Socioeconomic Status | | |  | 0.667 |  |  | 0.241 |
|  | Lower | 21(47.7) | 23(52.3) |  | 44(10.7) | 369(89.3) |  |
|  | Moderate | 84(40.9) | 121(59.1) |  | 205(13.0) | 1376(87.0) |  |
|  | Upper | 56(40.3) | 83(59.7) |  | 139(13.9) | 856(86.1) |  |
| Myopia | |  |  | 0.248 |  |  | **<0.001** |
|  | No | 265(42.7) | 357(57.3) |  | 622(28.6) | 1550(71.4) |  |
|  | Yes | 431(45.6) | 512(54.4) |  | 943(33.5) | 1876(66.5) |  |
| SE (D) | | -0.75 [-1.75, 0.00] | -0.50[-1.50,0.00] | **0.038** | -0.50[-1.50,0.00] | -0.50[-1.75,0.00] | 0.850 |
| Nutrition | |  |  | **0.003** |  |  | **<0.001** |
|  | Normal | 422(42.4) | 594(57.6) |  | 1016(33.3) | 2017(66.7) |  |
|  | OW | 134(47.5) | 148(52.5) |  | 282(28.7) | 697(71.3) |  |
|  | OB | 140(48.6) | 127(44.4) |  | 267(27.3) | 712(72.7) |  |
| BMI (kg/m^2^) | | 17.57 [15.60, 20.45] | 16.44[14.84,18.96] | **<0.001** | 16.93[15.15,19.82] | 17.74[15.64,21.07] | **<0.001** |
| Comorbidity of myopia and OWOB | | | | **0.038** |  |  | 0.865 |
|  | No | 521(42.9) | 690(57.1) |  | 1211(31.5) | 2642(68.5) |  |
|  | Yes | 175(49.4) | 179(50.6) |  | 354(31.1) | 784(68.9) |  |

Note: SE: spherical equivalent; BMI: body mass index; OWOB: overweight and obesity.

C.2.1 Adoption Dimension Methods

The study evaluated school and staff representativeness in the intervention. School representativeness was assessed by comparing participating schools' characteristics (public/private status, 9-year consistent education model, and student enrollment size) with regional/national benchmarks. Staff representativeness was determined by analyzing demographics of participating school physicians/teachers (age, sex, profession, and years of experience). School-level data were obtained from district education bureau records, including school type, enrollment size, and administrative affiliation.

Staff characteristics were collected through baseline questionnaires covering age, gender, qualifications, and professional tenure. National reference data came from the Ministry of Education's 2023 statistics. Descriptive analyses (percentage distributions) compared these characteristics between study participants and reference populations.

C.2.2 Adoption Dimension Results

A total of 36 schools were recruited, representing a balanced distribution across the three target districts (Dongcheng, Tongzhou, and Changping) with 12 schools (33.3%) from each region. The sample included equal numbers of primary and junior high schools (18 each), all of which were public institutions. Among these, 12 schools (33.3%) followed the 9-year consistent education model, while the majority (55.6%) had medium-sized student populations ranging from 1,000 to 3,000 students. This distribution ensured broad representation of different school types and sizes within Beijing's urban and suburban areas. See Figure C2.

Figure C2 Characteristic distribution of schools participating in this research project

Implementation relied on school-based personnel, with 123 staff members participating in program delivery. The implementing team was predominantly female (75.6%) with an average age of 41.3 years (±8.75), reflecting the typical demographic composition of school health personnel in China. Classroom teachers formed the largest professional group (48.8%), followed by school medical staff (13.8%) and physical education teachers (11.4%). Most participants held bachelor's degrees (64.4%) and had substantial work experience, with 39.3% possessing over 20 years of service. See Figure C3.

Figure C3 Characteristic distribution of teachers participating in this research project

C.3.1 Implementation Dimension Methods

Fidelity was measured as the percentage of manual-specified interventions completed, while protocol adaptations documented any modifications to intervention content or delivery methods. Cost analyses included total expenditure, per-participant cost (total cost/participants), incremental per-participant cost (intervention-group per-participant cost minus control-group per-participant cost), and descriptive cost per 1-percentage-point absolute difference in incident comorbidity.

Implementation data were extracted from project work logs and WeChat mini-program backend records, with protocol deviations and rationales identified from activity documentation. Expenditure records provided cost data. Quantitative analyses employed means, standard deviations, and percentages to assess fidelity and costs, with qualitative descriptions of protocol adaptations.

C.3.2 Implementation Dimension Results

Core school-based activities achieved high completion rates, with 100% of planned health education lectures and environmental modifications being implemented across all 18 intervention schools. This included the distribution of educational materials and the organization of both online and offline health lectures. However, family engagement components showed lower participation rates, only 60% of weekly evidence-based health tips were successfully disseminated to parents via school physicians and homeroom teachers.

The digital health tracking system revealed particularly limited uptake, as merely 594 participants engaged with the lifestyle monitoring features, submitting an average of 18.6 health behavior records over the intervention period. Implementation challenges emerged primarily in components requiring active family participation. While 183 students (7.1% of the intervention group) uploaded growth monitoring data through the digital platform, the average of 5.1 submissions per participant fell short of expected engagement levels. Similarly, only 132 families utilized the health diary function, with most records coming from a small subset of highly engaged participants. These patterns suggest significant barriers to sustained family involvement.

Figure C4 Schematic diagram of the page of the WeChat Applet

Project records indicate that implementation adjustments primarily focused on strengthening communication with school medical staff to improve intervention delivery, rather than modifying core program content.

The total cost of the project was 998,000 yuan, and the per-participant cost was 199.96 yuan. Costs were calculated based on the number of participants randomized at baseline. The total cost of the control group was 211,500 yuan, with an average cost of 88.13 yuan per participant, and the total cost of the intervention group was 786,500 yuan, with an average cost of 303.55 yuan per participant. The incremental per-participant cost was 215.43 yuan. Taking incident comorbidity as the outcome measure, the descriptive cost was 430.85 yuan per 1-percentage-point absolute difference in incident comorbidity, based on incidences of 8.7% in the intervention group and 9.2% in the control group (absolute difference, 0.5 percentage points). See Table C2.

Table C2. Implementation cost analysis

| **Cost Category** | **Amount (yuan)** | **Per Participant (yuan)** |
| --- | --- | --- |
| Total Program Costs | 998,000 | 199.96 |
| Intervention Group Costs | 786,500 | 303.55 |
| Control Group Costs | 211,500 | 88.13 |
| Incremental per-participant cost | — | 215.43 |
| Descriptive cost per 1-percentage-point absolute difference in incident comorbidity | - | 430.85 |

C.4.1 Maintenance Dimension Methods

After the intervention study concluded, sustainability questionnaires based on the Program Sustainability Assessment Tool (PSAT) were administered to participating school physicians, teachers, staff from health centers, and project team members. The PSAT was developed by the Center for Public Health Systems Science at Washington University in the U.S. and assesses sustainability across eight dimensions according to a sustainability framework: environmental support, funding stability, partnerships, organizational capacity, program evaluation, program adaptation, communications, and strategic planning (see Table C3). The short-form PSAT consists of 24 items, with three questions per sustainability dimension, and is suitable for scoring sustainability in public health, social service, and education programs. Respondents rated each item on a 1-7 scale (with “NA” option), where 1 indicates least alignment and 7 indicates full alignment with the statement.

Table C3 PSAT Framework Evaluation Dimensions

| Dimension | Definition |
| --- | --- |
| Environmental Support | Whether the program receives internal and external support |
| Funding Stability | Whether the program has established a sustainable funding base |
| Partnerships | Whether strong relationships have been formed with other agencies/community |
| Organizational Capacity | Whether there is internal support and resources to effectively manage the program |
| Program Evaluation | Whether regular effectiveness evaluations and data feedback occur during implementation |
| Program Adaptation | Whether the program can be adapted in response to changing needs or circumstances while maintaining its core components |
| Communications | Whether there is communication about the program with participants and the public |
| Strategic Planning | Whether the program has clear long-term strategies and goal planning |

In this tool, “program” refers to formally organized activities intended to continue over time, defined in this study as the Beijing Integrated Intervention for Myopia and Obesity Comorbidity in Children and Adolescents. “Organization” refers to departments/institutions implementing the program, here defined as the project team, district health centers, and collaborating medical institutions. “Community” refers to stakeholders who may benefit from or guide the program (including local residents, organizational leaders, policymakers), not specific towns/communities; here defined as beneficiaries (participating schools, children, parents) and guiding parties (project team, district health centers, and leadership of collaborating medical institutions).

Reliability testing of the scale showed an overall Cronbach's α coefficient of 0.933, indicating good reliability. Exploratory factor analysis for validity revealed KMO values of 0.625 (environmental support), 0.624 (funding stability), 0.603 (partnerships), 0.578 (organizational capacity), 0.722 (program evaluation), 0.631 (program adaptation), 0.610 (communications), and 0.610 (strategic planning).

Mean and standard deviation were used to describe overall and dimensional PSAT scores, with percentage stacked bar charts displaying response distributions for each item.

C.4.2 Maintenance Dimension Results

The overall sustainability score averaged 6.10±1.45 , indicating moderate potential for long-term maintenance. Notably, program evaluation capacity scored highest (6.58±0.80), reflecting strong monitoring systems, while funding stability emerged as the weakest dimension (4.41±2.28), highlighting significant concerns about financial continuity. Intermediate scores were observed for environmental support (6.55±0.85) and partner engagement (6.38±1.17), suggesting adequate but improvable infrastructure for sustained implementation. See Figure C5.

Figure C5 The mean score of each dimension of maintenance
